# Sedation practices for routine gastrointestinal endoscopy: a systematic review of recommendations

**DOI:** 10.1186/s12876-020-01561-z

**Published:** 2021-01-07

**Authors:** Fahima Dossa, Olivia Megetto, Mafo Yakubu, David D. Q. Zhang, Nancy N. Baxter

**Affiliations:** 1 Division of General Surgery, Department of Surgery, University of Toronto, Toronto, ON Canada; 2 Institute of Health Policy, Management, and Evaluation, Dalla Lana School of Public Health, University of Toronto, Toronto, ON Canada; 3 Ontario Health, Cancer Care Ontario, Toronto, ON Canada; 4 Melbourne School of Population and Global Health, University of Melbourne, Melbourne, VIC Australia

**Keywords:** Endoscopy, digestive system, Sedatives, Guideline

## Abstract

**Background:**

Sedation is commonly used in gastrointestinal endoscopy; however, considerable variability in sedation practices has been reported. The objective of this review was to identify and synthesize existing recommendations on sedation practices for routine gastrointestinal endoscopy procedures.

**Methods:**

We systematically reviewed guidelines and position statements identified through a search of PubMed, guidelines databases, and websites of relevant professional associations from January 1, 2005 to May 10, 2019. We included English-language guidelines/position statements with recommendations relating to sedation for adults undergoing routine gastrointestinal endoscopy. Documents with guidance only for complex endoscopic procedures were excluded.

We extracted and synthesized recommendations relating to: 1) choice of sedatives, 2) sedation administration, 3) personnel responsible for monitoring sedated patients, 4) skills and training of individuals involved in sedation, and 5) equipment required for monitoring sedated patients. We assessed the quality of included documents using the Appraisal of Guidelines for Research & Evaluation (AGREE) II tool.

**Results:**

We identified 19 guidelines and 7 position statements meeting inclusion criteria. Documents generally agreed that a single, trained registered nurse can administer moderate sedation, monitor the patient, and assist with brief, interruptible tasks. Documents also agreed on the routine use of pulse oximetry and blood pressure monitoring during endoscopy. However, recommendations relating to the drugs to be used for sedation, the healthcare personnel capable of administering propofol and monitoring patients sedated with propofol, and the need for capnography when monitoring sedated patients varied. Only 9 documents provided a grade or level of evidence in support of their recommendations.

**Conclusions:**

Recommendations for sedation practices in routine gastrointestinal endoscopy differ across guidelines/position statements and often lack supporting evidence with potential implications for patient safety and procedural efficiency.

## Background

Endoscopy is frequently used in the diagnosis and treatment of gastrointestinal (GI) disorders. However, patient fear and anxiety related to the anticipated discomfort of the procedure can limit willingness to undergo endoscopy and, in some cases, affect the endoscopist’s ability to successfully complete the procedure [1–3]. Sedation prior to and during endoscopy can decrease patient anxiety and discomfort, and improve the quality of the endoscopic procedure [4, 5].

The level of sedation targeted for GI endoscopy dictates the need for additional personnel and equipment. Moderate sedation, commonly provided through a combination of an intravenous benzodiazepine and opioid, refers to a level of sedation where patients remain responsive to verbal commands with or without the need for light tactile stimulation [6]. Patients sedated to this level are at risk of entering a deeper state of sedation where they become difficult to rouse without stimulation. Deep sedation with propofol, in contrast, refers to a level where patients require repeated or painful stimulation to elicit a response [6]; these patients are at risk of entry into general anesthesia, rendering them unconscious and potentially incapable of protecting their airway. Given the additional risk, deep sedation can be resource intensive, requiring additional personnel and equipment for monitoring [7]. Whether deep sedation with propofol provides additional benefits when compared with moderate sedation with midazolam is debated [8, 9].

Sedation practices vary considerably across jurisdictions [7, 10–12]. In an international study at 21 centres across 11 countries, Froehlich et al. [7] found large differences in the types of sedatives used, the individuals responsible for administering sedation, the number of staff members present for sedated colonoscopy, and the equipment used to monitor sedated patients. Differences in sedation practices and standards for monitoring sedated patients can have important implications for the safety of the procedure. Too few or inexperienced personnel, particularly when deep sedation is used, can put patients at risk for serious cardiovascular complications. However, increased personnel and monitoring equipment comes at the cost of negatively affecting the efficiency of the procedure, potentially limiting access to endoscopy. The objective of this review was to identify and synthesize recommendations from existing guidelines and position statements on the administration of sedation and appropriate monitoring of patients undergoing routine GI endoscopy.

## Methods

### Overview

The protocol for this review was developed according to the Preferred Reporting Items for Systematic Review and Meta-Analysis Protocols (PRISMA-P) checklist [13] and was registered with PROSPERO (CRD: 42019141076). This study aimed to synthesize existing recommendations for sedation practices for routine GI endoscopy in the following areas:

1. Classes of drugs recommended for sedation in routine GI endoscopy
2. Healthcare professionals capable of administering sedation
3. Healthcare professionals responsible for monitoring sedated patients
4. Required skills and training for individuals involved in sedation
5. Equipment required for monitoring sedated patients

This study is reported in accordance with PRISMA guidelines [14].

### Search strategy

We searched PubMed from January 1, 2005 to May 10, 2019 to identify guidelines and position statements. The search was developed by a senior information specialist; terms included variations of “endoscopy,” “colonoscopy,” “gastroscopy,” and “guidelines.” We also searched guideline databases, including the Standards and Guidelines Evidence Directory of Cancer Guidelines, the Agency for Healthcare Research and Quality National Guideline Clearing House, the National Institutes for Health and Care Excellence, the International Guideline Library, and the Canadian Medical Association InfoBase, over the same time-period, with the search terms, “colonoscopy,” “gastroscopy,” “endoscopy,” and “sedation.” Finally, we searched the websites of relevant professional associations over the same time-period using identical search terms. A complete list of the sources searched and the search strategies is provided in Additional file 1: Appendix 1. All searches were limited to English-language documents.

### Selection criteria

We included guidelines and position statements relating to the use of sedation among adult patients (≥18 years) undergoing routine GI endoscopy, defined as elective gastroscopy and/or colonoscopy. We excluded documents limited to pediatric populations or pregnant women; documents focused solely on advanced procedures (e.g. endoscopic retrograde cholangiopancreatography [ERCP], endoscopic ultrasound [EUS], endoscopic mucosal resection [EMR], endoscopic submucosal dissection [ESD]) or emergency procedures; and those providing recommendations for procedural sedation not specific to GI endoscopy (unless the non-specific recommendations were endorsed by a gastroenterological society). Commentaries, editorials, systematic reviews without accompanying guidelines, primary research studies, and non-English-language documents were excluded.

Two independent reviewers assessed all titles and abstracts for eligibility. All disagreements were resolved through discussion. Similarly, full text documents were independently assessed for eligibility by two independent reviewers, with consensus achieved through discussion.

### Data extraction

For each included document, we extracted descriptive information (document developer, title, year of publication, jurisdiction/location), recommendations pertaining to each study aim and the corresponding evidentiary base and grade, the endoscopic procedure(s) the recommendation applied to, and whether the recommendation applied to a specific level of sedation or type of sedative agent. We developed and used standardized electronic data extraction forms on the DistillerSR web-based platform (Evidence Partners, Ottawa, Ontario, Canada) to facilitate data extraction. Two independent reviewers extracted data from all included documents and all discrepancies were resolved through discussion.

### Quality assessment

We assessed the quality of all included documents using the Appraisal of Guidelines for Research & Evaluation (AGREE) II instrument, a validated tool to assess the quality and reporting of practice guidelines [15]. The instrument contains 23 items organized into six domains: scope and purpose, stakeholder involvement, rigor of development, clarity of presentation, applicability, and editorial independence. Each item was rated on a 7-point scale (1-strongly disagree to 7-strongly agree) by two independent reviewers; discrepancies were resolved by discussion. Domain scores were calculated by summing the scores of the individual items in a domain and by scaling the total as a percentage of the maximum possible score for that domain. In accordance with the AGREE II User Guide [15], the study team prioritized the rigor of development domain; documents scoring ≥60% were considered to have appropriately addressed this domain [16, 17].

### Data synthesis

For each objective, we synthesized recommendations and presented results by level of sedation (i.e. moderate or deep), the administration of propofol, or sedation practice in general (i.e. not tied to a specific level of sedation or propofol administration). We identified similarities, differences, and gaps in recommendations within these groups for each study objective.

### Patient and Public Involvement

Patients were not involved in the design of this study.

## Results

**Fig. 1:**
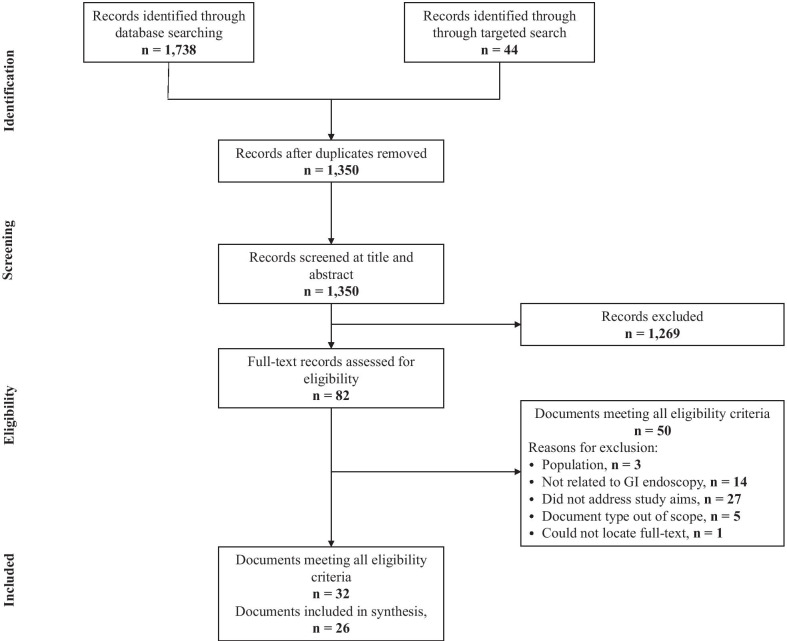
Study flow diagram

The study flow diagram is presented in Fig. 1. We identified 1350 citations through database and targeted searching. Following title and abstract screening, the full-text records of 82 documents were assessed for eligibility. Thirty-two documents met all inclusion criteria (Additional file 1: Appendix 2) [18–49]. Five of these documents were updates of previously published guidelines [18, 23, 34] or position statements [43, 45] – only the most recent versions were included [20, 24, 27, 44, 46]; 2 citations were considered companion documents for each other and treated as a single result [38, 39]. Therefore, 26 documents were included in the final synthesis, including 19 guidelines [19–22, 24–33, 35–37, 39, 40] and 7 position statements [41, 42, 44, 46–49].

### Characteristics of included documents

A summary of the characteristics of the included documents is provided in Supplemental Table 1. Though most documents addressed GI endoscopy procedures in general, four documents specifically addressed colonoscopy [33, 35, 39, 40]. Some organizations developed more than one document addressing different topics related to GI endoscopy practice, including 7 documents from the American Society of Gastrointestinal Endoscopy (ASGE) [21, 26–28, 32, 42, 49], 2 documents from the Spanish Society of Gastrointestinal Endoscopy (SSGE) [31, 33], and 2 documents from the Society of Gastroenterology Nurses and Associates (SGNA) [41, 46].

### Quality Assessment

Scores on the AGREE II domains were generally low (Table 1). Among guidelines, the highest scoring domains were scope and purpose (average score = 53%) and clarity of presentation (average score = 47%); the lowest scoring domain was applicability (average score = 9%). Position statements had similar scores for the clarity of presentation domain (average score = 45%) but lower scores in the scope and purpose domain (average score = 27%). The majority (*n* = 22) of included documents scored < 60% in the rigor of development domain. Of these, 15 were guidelines [19–22, 25–33, 35, 37] and 7 were position statements [41, 42, 44, 46–49]. Only 9 documents [24, 26, 27, 29, 31, 33, 36, 40, 49] provided a grade or level of evidence in support of their recommendations, making it difficult to assess the evidentiary base for the reported recommendations. When used, recommendation and evidence grading systems varied across documents, making cross-document comparisons challenging.

**Table 1:** Quality assessment of included documents using the AGREE II tool

Document developer	Scope and purpose (%)	Stakeholder involvement (%)	Rigor of development (%)	Clarity of presentation (%)	Applicability (%)	Editorial independence (%)
*Guidelines (n = 19)*						
ASGE (2006) [28]	61	47	1	14	2	0
BSG (2006) [29]	58	44	21	22	2	0
FSDE (2006) [35]	6	14	15	22	6	0
AGA (2007) [22]	36	36	10	64	4	0
ASGH (2007) [37]	11	0	7	17	0	42
SAGES (2009) [30]	42	22	4	11	4	0
ASGE (2010) [32]	53	39	27	61	0	0
GESA (2014) [20]	53	25	1	33	0	0
DSRPGSA (2011) [19]	69	28	2	28	10	0
SSGE (2012) [33]	58	28	43	61	10	0
EC (2012) [40]	67	58	82	78	44	100
ASGE (2013) [26]	64	50	30	36	0	42
ASGE (2014) [21]	56	31	11	58	2	33
CCO (2013, 2014) [38, 39]	94	78	76	69	38	100
SSGE (2014) [31]	36	33	20	64	15	0
ESGE & ESGENA (2015) [24]	58	42	66	75	10	38
GSGDMD (2014) [36]	69	56	73	72	6	83
ASGE (2018) [27]	36	36	30	61	15	0
JAG (2019) [25]	78	61	42	56	8	100
*Position Statements (n = 7)*						
CAG (2008) [47]	39	3	9	53	2	0
SGNA (2008) [41]	17	11	4	19	0	0
ASGE (2009) [49]	28	44	48	50	2	58
ASGE, ACG, & AGA (2012) [42]	11	14	13	28	13	0
CSGNA (2015) [44]	19	17	2	44	0	0
SGNA (2016) [46]	33	25	6	36	13	0
ISDE (2017) [48]	44	11	31	83	25	50

*ACG* American College of Gastroenterology, *AGA* American Gastroenterological Association, *ASGE* American Society for Gastrointestinal Endoscopy, *ASGH* Austrian Society of Gastroenterology and Hepatology, *BSG* British Society of Gastroenterology, *CAG* Canadian Association of Gastroenterology, *CCO* Cancer Care Ontario, *CSGNA* Canadian Society of Gastroenterology Nurses and Associations, *DSRPGSA* Danish Secretariat for Reference Programmes for Gastroenterology, Surgery and Anaesthetics, *EC* European Commission, *ESGE* European Society of Gastrointestinal Endoscopy, *ESGENA* European Society of Gastrointestinal Endoscopy Nurses and Associates, *FSDE* French Society of Digestive Endoscopy, *GESA* Gastroenterological Society of Australia, *GSGDMD* German Society for Gastroenterology, Digestive and Metabolic Diseases, *ISDE* Italian Society of Digestive Endoscopy, *JAG* Joint Advisory Group, *SAGES* Society of American Gastrointestinal and Endoscopic Surgeons, *SGNA* Society of Gastroenterology Nurses and Associates, *SSGE* Spanish Society of Gastrointestinal Endoscopy

### Recommended agents for sedation

Twelve documents provided guidance on the choice of sedatives for GI endoscopy (Table 2) [19, 21, 22, 24, 26, 27, 31, 33, 35, 36, 40, 47].

**Table 2:** Summary of recommendations for sedative agents

Subject	Document	Recommendation or Statement (Quote)	Strength	Level of evidence
**Moderate sedation**				
Use midazolam over other benzodiazepines	GSGMD [36]	If benzodiazepines are used for sedation because of their stronger amnestic effect, we suggest that midazolam be preferred to diazepam because of its shorter half-life	B	2a
	SSGE [31]	When benzodiazepines are used, midazolam is recommended	B	2++
Moderate sedation provides high satisfaction for patients and physicians	SSGE [31]	Moderate sedation using currently available drugs for routine endoscopic procedures (colonoscopies and gastroscopies) is highly satisfactory for patients and physicians alike given their low risk for adverse events	A	1-
**Depth of sedation/choice of agent**				
Moderate sedation/benzodiazepines adequate	SSGE [31]	Moderate sedation using currently available drugs for routine endoscopic procedures (colonoscopies and gastroscopies) is highly satisfactory for patients and physicians alike given their low risk for adverse events	A	1-
		For non-complex diagnostic or therapeutic gastroscopy and colonoscopy superficial sedation suffices	A	1+
	ESGE [24]	Simple endoscopic procedures can be performed with moderate sedation, maintaining a high degree of patient satisfaction. Prolonged or complex procedures (e.g. EUS, ERCP) are frequently performed under deep sedation	Strong	High
	CAG [47]	It should be recognized that adequate sedation can usually be achieved with a combination of opioids and benzodiazepines. As such, there is no mandate for endoscopists to switch to propofol, particularly because most operators have considerable experience administering standard agents	–	–
	ASGE [27]	We recommend that the combination of an opioid and benzodiazepine is a safe and effective regimen for achieving minimal to moderate sedation for upper endoscopy and colonoscopy in patients without risk factors for sedation-related adverse events	–	High
Deep sedation/propofol preferred	GSGMD [36]	Because of data on efficacy, recovery, and complications, we suggest that propofol should be preferred to midazolam	B	2b
	SSGE [33]	Literature data available on effectiveness, recovery issues, and complications seem to favor the use of propofol over benzodiazepines	B	2b
	SSGE [31]	Propofol is an ideal drug to provide sedation for endoscopic examinations For complex or prolonged procedures (ERCP, EUS, etc.) deep sedation is to be preferred	A	1+
	FSDE [35]	All patients undergoing a colonoscopy must be offered a general anesthesia. However, an examination without general anesthesia is conceivable for patients who have been told about the potential plan.	–	–
Individualize	GSGMD [36]	We recommend that the type and intensity of the sedation and the drug used should be selected according to the type of intervention and the patient’s ASA grade and individual risk profile	A	5
	ASGE [21]	The choice of specific sedation agents and the level of sedation targeted should be determined on a case-by-case basis by the endoscopist in consultation with the patient	–	–
	EC [40]	Because there is no clear benefit from a particular approach and for practical reasons, it is recommended that policies on the use of sedation should be adopted according to protocols based on national or pan-European guidelines, and must take into account historical context, the impact on the patient experience, and cost	B	I
	ASGE [27]	We suggest that endoscopists use propofol-based sedation (endoscopist-directed or anesthesia-provider administered) when it is expected to improve patient safety, comfort, procedural efficiency, and/or successful procedure completion	–	Low
	SSGE [31]	Sedation level and drug type depend on procedure characteristics, individual patient-related factors, patient preferences, and need for patient cooperation	D	4
**Propofol sedation**				
Delivery	GSGMD [36]	We suggest that propofol should be administered by intermittent bolus administration	B	1b
	ESGE [24]	We recommend administering propofol through intermittent bolus infusion or perfusor system, including target-controlled infusion (TCI), and consideration of patient-controlled sedation (PCS) in particular settings	Strong	High
Avoid concomitant use of pharyngeal anesthesia	ESGE [24]	We do not suggest using pharyngeal anesthesia during propofol sedation for upper GI endoscopy	Weak	Moderate
Use propofol monotherapy	ESGE [24]	We suggest propofol monotherapy except in particular situations. In some situations, low dose midazolam premedication might be beneficial to facilitate intravenous line placement and to reduce the need for propofol. Such situations include patients with high anxiety potential, long-lasting procedures in patients with a known important need for sedatives, and patients with limited left ventricular function or with previous pronounced hypotension following propofol administration	Weak	High
	GSGMD [36]	We suggest that a combination of propofol and midazolam should not be used	B	1b
	DSRPGSA [19]	Propofol is administered intravenously and should be used only as monotherapy	–	–
Consider use of balanced propofol administration	SSGE [31]	Midazolam administration before propofol allows to reduce dosage and adverse effects, particularly hypotension in cardiac patients or in hypovolemia, but recovery is delayed	B	1+
Special populations	GSGDM [36]	Propofol may be considered for sedation in elderly populations	Statement	1b
	GSGMD [36]	We recommend that propofol should be used for sedation of patients with hepatic encephalopathy. Benzodiazepines should not be used in patients with hepatic encephalopathy	A	1b
**Sedation practice in general**				
Offering sedation	GSGMD [36]	We recommend that sedation should be offered to every patient before endoscopy. The advantages and disadvantages should be discussed in detail	A	5
	GSGMD [36]	We suggest that, on principle, simple endoscopic examinations can be performed without sedation	Statement	2b
Use of adjunctive agents	GSGMD [36]	We suggest that opioids, ketamines, inhalational anesthetics, and neuroleptics should not be used as monotherapeutics for sedation in endoscopy	B	5
	GSGMD [36]	Nitrous oxide (laughing gas) may be considered for analgesia and sedation during colonoscopy; appropriate structural requirements must be met	Statement	1b
	AGA [22]	The majority of patients can be adequately sedated by using a combination of an opioid and benzodiazepine. The addition of an adjunctive agent in combination with conventional sedation drugs may be useful for the difficult-to-sedate patient	–	–
Titrating sedative doses in special populations	ASGE [26]	We recommend that lower initial doses of sedatives than standard adult dosing should be considered in the elderly and that titration should be more gradual to allow assessment of the full dose effect at each dose level	–	Moderate
	GSGMD [36]	Patients with higher ASA grade and/or older patients are at higher risk of sedation-related side effects (cardiorespiratory depression). We suggest that the dose of the sedative/analgesic used should be adjusted/reduced accordingly	B	2b

*AGA* American Gastroenterological Association, *ASGE* American Society for Gastrointestinal Endoscopy, *CAG* Canadian Association of Gastroenterology, *DSRPGSA* Danish Secretariat for Reference Programmes for Gastroenterology, Surgery and Anaesthetics, *EC* European Commission, *ESGE* European Society of Gastrointestinal Endoscopy, *FSDE* French Society of Digestive Endoscopy, *GSGDMD* German Society for Gastroenterology, Digestive and Metabolic Diseases, *SSGE* Spanish Society of Gastrointestinal Endoscopy

Two documents made recommendations about specific agents for moderate sedation. Both the German Society for Gastroenterology, Digestive and Metabolic Diseases (GSGMD) [36] and the SSGE [31] stated that midazolam is the preferred benzodiazepine for moderate sedation based on systematic reviews of cohort studies.

With respect to the administration of propofol, documents were not consistent in their recommendations. Documents from the GSGMD [36], European Society of Gastrointestinal Endoscopy (ESGE) & European Society of Gastrointestinal Endoscopy Nurses and Associates (ESGENA) [24], and the Danish Secretariat for Reference Programmes for Gastroenterology, Surgery, and Anaesthetics (DSRPGSA) [19] recommended propofol monotherapy. In contrast, a guideline from the SSGE [31] recommended that patients receiving propofol be pre-medicated with midazolam to reduce the total dose of and adverse events associated with propofol; the ESGE/ESGENA [24] guideline recommended pre-medication with midazolam only in select cases (Table 2). The GSGMD [36] recommended administration by intermittent boluses, whereas the ESGE/ESGENA [24] recommended intermittent bolus or perfusor systems, such as target-controlled or patient-controlled infusion systems. The ESGE/ESGENA guideline [24] also suggested against the use of pharyngeal anesthesia for patients undergoing upper GI endoscopy under propofol sedation.

There was no consensus across documents on the optimal sedating agents (i.e. benzodiazepine +/− opioid vs. propofol) or targeted depth of sedation (i.e. moderate vs. deep) (Table 2). Based on high-quality evidence, documents from the SSGE [31] and ESGE/ESGENA [24] stated that moderate sedation provides high patient satisfaction for GI endoscopy but that deep sedation is preferred for complex procedures (e.g. EUS, ERCP). Although propofol can be targeted to moderate sedation (e.g. with use of balanced propofol sedation, which combines propofol with a benzodiazepine and opioid [22, 27, 50]), documents from the ASGE [27] and the Canadian Association of Gastroenterology (CAG) [47] specifically stated that the combination of an opioid and benzodiazepine is adequate for routine endoscopy. In contrast, documents from the GSGMD [36] and SSGE [31, 33] expressed a preference for propofol over benzodiazepines. However, several documents, including guidelines from the GSGMD [36], ASGE [21, 27], SSGE [31], and European Commission (EC) [40], made recommendations for tailoring the agent and depth of sedation to the patient, generally based on low-quality evidence (Table 2). A document from the French Society of Digestive Endoscopy (FSDE) [35] stated that general anesthesia should be used for all patients undergoing colonoscopy; however, this document did not define general anesthesia, nor did it specify which agents were recommended for use.

### Personnel capable of administering sedation

Nineteen documents (14 guidelines [19–22, 24, 27, 29–33, 35–37] and 5 position statements [41, 44, 47–49]) provided recommendations regarding the types of healthcare professionals capable of administering sedation for routine GI endoscopy. These recommendations are summarized in Table 3. Few documents detailed the level of evidence or the strength of the recommendations (*n* = 6) [24, 27, 31, 33, 36, 49] (Supplemental Table 2).

**Table 3:** Summary of recommendations for individuals capable of administering sedation

Subject	Number of documents	Document developers	Comments
**Moderate sedation**			
Can be administered by a nurse who is directed by a physician	4	ASGE [21, 27, 32], SGNA [41]	–
Should be administered by a practitioner other than the endoscopist	1	GESA [20]	Trained medical/dental practitioner (with advanced life support skills)
**Deep sedation**			
Should be administered by an anesthesia professional	3	ASGE [21]	Anesthesiologist, Certified Registered Nurse Anesthetist (CRNA), or Anesthesiologist Assistant (as determined by institutional policies)
		GESA [20]	Anesthetist or other appropriately trained and credentialed medical specialist within his/her scope of practice
		SGNA [41]	Anesthesiologist
**Propofol**			
Should not be administered by nurses	3	CSGNA [44]	Not within scope of practice
		GESA [20]	Intravenous anesthetics should be administered by a second medical or dental practitioner
		BSG [29]	–
Non-anesthesiologist propofol administration can be considered	8	GSGMD [36]	Administered by a non-physician, who has sedation as their sole task, under the instruction of a physician can be considered
		DSRPGSA [19]	Can be administered by a nurse under direction of a non-anesthetist physician
		AGA [22]	Gastroenterologist-directed administration is safe
		SSGE [33]	Administration by non-anesthesiologist is safe
		SSGE [31]	Administration by endoscopist/trained nurse safe and may improve efficiency
		CAG [47]	Administration by endoscopists and/or trained endoscopy nurses is safe; anesthesiologist not required for low-risk patients
		ASGE [49]	Administration by non-anesthesiologists improves practice efficiency for healthy, low-risk patients undergoing routine GI endoscopy
		ISDE [48]	Administration by trained non-anesthesiologists is safe
An anesthesiologist should be readily available when non-anesthesiologist propofol sedation is used	2	DSRPGSA [19]	Must be in immediate vicinity
		SSGE [31]	Available within 5 min
Patient and procedure factors to consider when determining whether an anesthesiologist is required			
ASA class	7	ESGE [24], DSRPGSA [19], SSGE [31, 33], CAG [47], ISDE [48]	ASA ≥ III
		GSGMD [36]	ASA IV-V
Mallampati class or facial features	1	ESGE [24]	Mallampati class ≥3 Dysmorphic facial features or oral abnormalities (mouth opening < 3 cm, high arched palate, macroglossia, micrognathia)
Other factors suggestive of difficult intubation or ventilation	5	SSGE [31]	Short neck, sleep apnea
		ESGE [24]	Pharyngolaryngeal tumors, history of stridor, snoring, obstructive sleep apnea, neck or cervical spine abnormalities, tracheal deviation, advanced rheumatoid arthritis
		DSRPGSA [19]	BMI ≥35, non-compliance with fasting guidelines, respiratory assessment score ≥ 4
		CAG [47]	Difficulty anatomy for ventilation (obesity, thick neck)
		ISDE [48]	Difficult anatomy for ventilation (obesity, thick neck)
Patients with other high risk conditions	2	DSRPGSA [19]	Acute upper GI hemorrhage, sub-acute bowel obstruction/ileus, achalasia, sleep apnea, SpO2 < 95% with supplemental oxygen
		SSGE [31]	Chronic decompensated serious diseases
Long or complex procedures	5	DSRPGSA [19]	> 1 h
		SSGE [31]	Complex therapeutic procedures
		CAG [47]	Prolonged or high-risk interventional procedures
		ESGE [24]	Long-lasting procedures
		ISDE [48]	Long-lasting or high-risk interventional procedures
Other risk factors	3	ESGE [24]	Chronic narcotic use, intolerant to sedatives, difficult to sedate
		DSRPGSA [19]	Previous problems with anesthesia
		ISDE [48]	Uncooperative patients
**Sedation practice in general**			
The role of nurses in the administration of sedation	5	CSGNA [44]	Competent Registered Nurses can administer sedation when directed by a physician
		ASGH [37]	An individual must be present who is responsible for sedation administration (can be a trained assistant, nurse, member of the general medical staff, or anesthesiologist)
		ASGE [21]	Licensed practical nurses and unlicensed assistive personnel not qualified to administer sedation
		GESA [20]	Appropriately trained nurse may administer sedatives under direction of the physician
		SAGES [30]	Nurses administering sedation must work within their scope of practice
Intravenous sedation should be administered by an anesthesiologist	1	FSDE [35]	Non-anesthesiologist IV sedation should only be used in clinical trials
Patients and procedure factors to consider when determining whether an anesthesiologist is required			
ASA class	5	GSGMD [36]	≥III
		AGA [22], GESA [20], ASGE [27], SSGE [31]	IV-V
Mallampati class or facial features	2	GSGMD [36]	Mallampati grade 3 or 4, mouth opening < 2 cm, hyoid-to-chin distance < 4 cm
		SSGE [31]	Mallampati grade 4, mouth opening < 3 cm, decreased hyoid-chin distance, protruding incisors, macroglossia, gothic plate, tonsillar hypertrophy, retrognathia, micrognathia, trismus, severe dental malocclusion, dysmorphic face (Trisomy 21, Pierre-Robin sequence)
Other factors suggestive of difficult intubation or ventilation	5	GSGMD [36]	Craniofacial malformation; lingual, laryngeal, or hypopharyngeal tumor; severely restricted mobility of the cervical spine
		GESA [20]	Morbid obesity, significant obstructive sleep apnea, known or suspected difficult endotracheal intubation, potential for aspiration
		ASGE [27]	Anatomical variants portending increased risk for airway obstruction
		SSGE [31]	History of laryngeal stridor, sleep apnea, short thick neck, limited cervical extension, cervical spine conditions, trauma, severe tracheal deviation
		AGA [22]	Morbid obesity
Patients with other high risk conditions	3	GESA [20]	Elderly; severely limiting heart, cerebrovascular, lung, liver, or renal disease; acute GI bleeding; severe anemia
		ASGE [27]	Multiple medical comorbidities or at risk for airway compromise
		BSG [29]	Outflow obstruction or any serious form of cardiac or pulmonary compromise
Long or complex procedures	4	GSGMD [36]	Difficult endoscopic intervention
		AGA [22]	ERCP, stent placement in upper GI tract, EUS, complex therapeutic procedures (e.g. ESD, plication of the cardioesophageal junction, EGD with drainage of pseudocyst)
		ASGE [27]	Complex endoscopic procedures
		SSGE [31]	Urgent, prolonged, or therapeutically complex procedures
Other risk factors	5	AGA [22]	History of alcohol or substance abuse, pregnancy, neurological/neuromuscular disorders, uncooperative or delirious patients
		GESA [20]	Previous sedation-related adverse events
		ASGE [27]	Anticipated intolerance to sedatives
		SSGE [31]	Intolerance or allergy to standard sedatives
		BSG [29]	Severe learning difficulties, patients who have previously failed or are likely to fail sedation including alcoholic or drug addicted patients, poor venous access; uncooperative or phobic patients

*AGA* American Gastroenterological Association, *ASGE* American Society for Gastrointestinal Endoscopy, *ASGH* Austrian Society of Gastroenterology and Hepatology, *BSG* British Society of Gastroenterology, *CAG* Canadian Association of Gastroenterology, *CSGNA* Canadian Society of Gastroenterology Nurses and Associations, *DSRPGSA* Danish Secretariat for Reference Programmes for Gastroenterology, Surgery and Anaesthetics, *ESGE* European Society of Gastrointestinal Endoscopy, *FSDE* French Society of Digestive Endoscopy, *GESA* Gastroenterological Society of Australia; *GSGDMD* German Society for Gastroenterology, Digestive and Metabolic Diseases, *ISDE* Italian Society of Digestive Endoscopy, *SAGES* Society of American Gastrointestinal and Endoscopic Surgeons, *SGNA* Society of Gastroenterology Nurses and Associates, *SSGE* Spanish Society of Gastrointestinal Endoscopy

Five documents, from 3 organizations, provided recommendations relevant to the administration of moderate sedation [20, 21, 27, 32, 41]. Documents from the ASGE [21, 27, 32] and SGNA [41] supported nurse-administered moderate sedation with supervision from a physician. A guideline from the GESA [20], however, stated that an “appropriately trained medical practitioner,” who is not the endoscopist, is required to administer intravenous sedation.

Recommendations for administration of deep sedation were provided in documents from the ASGE [21], Gastroenterological Society of Australia (GESA) [20], and SGNA [41]. Although all three documents recommended that an anesthesia professional be involved in the administration of deep sedation, documents varied in the strength of their recommendations and the suggested personnel – the SGNA recommended that involvement of an anesthesiologist be considered for patients undergoing deep sedation [41]; the ASGE suggested that anesthesia professionals could include an anesthesiologist, a certified registered nurse anesthetist, or an anesthesiology assistant, depending on institutional policies [21]; and the GESA stated that an anesthesiologist or other appropriately trained and credential medical specialist must be present when deep sedation is used [20].

Recommendations specific to the administration of propofol were provided in 13 documents [19, 20, 22, 24, 27, 29, 31, 33, 36, 44, 47–49] and varied considerably. Documents from the CSGNA [44], GESA [20], and British Society of Gastroenterology (BSG) [29] did not support nurse-administered propofol sedation; the GESA [20] and BSG [29] further recommended that propofol be administered by an anesthesiologist or a second, appropriately trained, medical practitioner who is not the endoscopist [20]. In contrast, documents from the ESGE [24], DSRPGSA [19], ASGE [49], and Italian Society of Digestive Endoscopy (ISDE) [48] specifically focused on non-anesthesiologist administered propofol (NAAP) sedation and 5 additional documents made recommendations regarding cases in which NAAP sedation would be appropriate [22, 31, 33, 36, 47]. These documents generally stated that NAAP sedation is safe in appropriately selected patients [22, 31, 33, 47, 48] and may improve efficiency of the endoscopy unit [31, 49]. The GSGMD [36], DSRPGSA [19], SSGE [31], and CAG [47] specifically stated that propofol administration by nurses, under the direction of physicians, is safe for low-risk patients and documents from the SSGE [31, 33], ASGE [49], and ISDE [48] stated that involvement of an anesthesiologist for low-risk patients undergoing propofol sedation is not cost-effective. To ensure safety, two documents recommended that an anesthesiologist be readily available when NAAP sedation is used [19, 31]. Furthermore, most documents discussing propofol sedation provided recommendations for circumstances that would necessitate administration of propofol by an anesthesiologist. Important factors in the decision of whether to involve an anesthesiologist included the patient’s American Society of Anesthesiologists (ASA) class [19, 24, 31, 33, 36, 47, 48]; Mallampati class, presence of facial abnormalities, or other factors suggestive of difficult intubation or ventilation [19, 24, 31, 47, 48]; patients with other high risk medical conditions [19, 31]; long and complex procedures [19, 24, 31, 47, 48]; and other risk factors, including individuals with previous problems with sedation, uncooperative patients, and chronic narcotic users (Table 3) [19, 24, 48].

Similar recommendations were made for the involvement of an anesthesiologist for sedation practice in general (Table 3). Unique recommendations relating to sedation practice in general included a recommendation from the ASGE [21] that licensed practical nurses and unlicensed assistive personnel are not qualified to administer sedation and a recommendation from the FSDE [35] that non-anesthesiologist intravenous sedation (not otherwise specified) not be used outside of clinical trials.

### Personnel Responsible for Monitoring Sedated Patients

Recommendations for the healthcare personnel required for monitoring sedated patients were discussed in 17 documents (12 guidelines [19–22, 24, 25, 27, 30–32, 36, 37] and 5 position statements [41, 44, 46–48]); few documents detailed the level of evidence or the strength of these recommendations (*n* = 3) [24, 31, 36] (Supplemental Table 3).

For moderately sedated patients, there was consensus among documents from the American Gastroenterological Association (AGA) [22], ASGE [21, 27, 32], SGNA [41], and the Society of American Gastrointestinal and Endoscopic Surgeons (SAGES) [30] that a single nurse is capable of both monitoring a moderately sedated patient and performing brief, interruptible tasks. A position statement from the Canadian Society of Gastroenterology Nurses and Associates (CSGNA) [44] recommended that two health professionals be present in the endoscopy suite when moderate sedation is being used but did not define who these health professionals could be. There was also consensus among documents from the ASGE [21, 32] and SGNA [41] that a second assistant be available to assist the endoscopist in complex procedures (e.g. difficult polypectomy) [21, 32, 41] or severely ill patients [41], allowing the nurse administering sedation to focus on monitoring the patient. Guidelines from the ASGE [21, 32] stated that these second assistants could be registered nurses, licensed practical nurses, or unlicensed assistive personnel.

Five documents provided recommendations for patients undergoing deep sedation. Documents from the AGA [22], ASGE [21, 32], and SGNA [41] consistently recommended that when deep sedation is used, the individual monitoring the sedated patient should not have any other responsibilities. This necessitates an additional individual to assist the endoscopist with technical aspects of the procedure. Two guidelines suggested that an anesthesia professional be present to monitor the deeply sedated patient [21, 37]. A guideline from the Austrian Society of Gastroenterology and Hepatology (ASGH) [37] suggested involving an anesthesiologist for patients who may require endotracheal intubation. The ASGE [21] recognized that many institutions require an anesthesia professional for administration of deep sedation and recommended that this individual also monitor the patient during the procedure.

Two documents [20, 25] made recommendations for monitoring patients under general anesthesia; however, recommendations in these documents differed. A guideline from Joint Advisory Group on Gastrointestinal Endoscopy (JAG) [25] recommended that an anesthesiologist be present to monitor patients under general anesthesia, whereas a guideline from GESA [20] recommended that both an anesthesiologist and an individual dedicated to assisting the anesthesiologist be present.

Documents providing recommendations for monitoring patients sedated with propofol were generally in agreement that use of propofol sedation requires an individual dedicated to monitoring the patient who has no other responsibilities, necessitating a second individual to assist the endoscopist with the procedure [19, 24, 27, 36, 47, 48]. However, a document from the SSGE [31] recommended that patient and procedure complexity be considered when determining whether an individual dedicated to monitoring sedation is needed. This document stated that basic endoscopic procedures on ASA class I-II patients do not require dedicated sedation staff but that complex therapeutic procedures or procedures performed on higher risk individuals (ASA > III) be staffed by individuals solely dedicated to monitoring the sedated patient. Two documents further recommended that a physician be present and available from the time of propofol administration to when the patient wakes up [19] or is ready for discharge [27], but were not specific as to who this physician could be.

Documents providing guidance for sedation practices in general made a range of recommendations (Supplemental Table 3), including that a minimum of one nurse is required for endoscopy with sedation [22, 46]; nurses are capable of monitoring sedating patients and performing brief, interruptible tasks [46]; sedated endoscopy requires an individual solely dedicated to monitoring the sedated patient [20, 36]; additional staff are required for complex procedures or endoscopy performed on high-risk patients [30, 36, 37, 44, 46]; and when sedation is provided by anesthesia personnel, an individual responsible for assisting the endoscopist, and possibly the anesthesia professional, is needed [20, 21, 32, 44, 46]. Uniquely, the CSGNA [44] stated that a second Registered Nurse, Licensed Practical Nurse, or Registered Practical Nurse is required for “therapeutic” procedures; however, this document did not define the procedures considered to be therapeutic.

### Skills and training required to administer sedation and monitor sedated patients

Seventeen documents (12 guidelines [19–22, 24, 27, 28, 30, 31, 33, 36, 37] and 5 position statements [41, 44, 47–49]) provided varying recommendations regarding the skills and training required for individuals involved in procedural sedation for endoscopy, often without a grade or level of evidence stated (Supplemental Table 4). Most recommendations for moderate sedation referred specifically to nurses. Recommendations included formal training in procedural sedation [44], knowledge of the sedatives used and their reversal agents [41, 44], an understanding of airway management [44], skills to rescue patients who enter deeper levels of sedation than intended [44], and the ability to manage other complications [41, 44]. Guidelines from the AGA [22] and ASGE [28] also recommended that physicians involved in these procedures be able to rescue patients from deeper levels of sedation than intended. The CSGNA [44] further recommended that endoscopy nurses working in hospitals have at least basic cardiac life support training and those working in private endoscopy clinics have advanced cardiac life support (ACLS) training; this document did not require ACLS training for nurses working within institutions with code response teams.

Documents addressing deep sedation similarly recommended knowledge of the medications used [41], the ability to rescue patients from a deeper level of sedation than intended (i.e. general anesthesia) [28], and skills in advanced airway management and the management of cardiorespiratory complications [22, 28, 41]. The SGNA specifically recommended that both nurses and physicians involved in deep sedation have skills in ACLS [41].

Prior to involvement in non-anesthesiologist administered propofol sedation, documents recommended formal training in propofol administration [19, 22, 24, 27, 47–49]. The ESGE [24] further stated that intensive care or anesthesia experience for the physician directing propofol sedation is desirable. Additional recommendations for propofol administration included basic resuscitation skills [19, 49], skills in managing complications [19, 27, 47], and skills in airway management [19, 27, 28, 47–49]. Documents from CAG [47], ASGE [27, 49], and ISDE [48] recommended ACLS training and the ESGE [24] recommended that if the individual administering propofol has ACLS training a life support team does not need to be rapidly available.

Recommendations for sedation practice not tied to a specific level of sedation were similar, including recommendations for formal training in sedation [20, 27, 30, 31, 36], knowledge of the agents being used [21, 22, 28, 31, 36, 37], the ability to recognize and manage complications and rescue patients from deeper than intended levels of sedation [20–22, 27, 28, 31, 37], skills in basic resuscitation [21, 22, 30, 31, 36] and airway management [20, 22, 31, 36], and ACLS training [20–22, 30, 33]. Uniquely, the GSGMD recommended that the physician responsible for sedation generally have intensive care medicine experience [36].

### Equipment required to monitor sedated patients

Equipment recommendations are summarized in Table 4. The equipment most consistently recommended for monitoring all sedated patients (regardless of level targeted or use of propofol) included non-invasive blood pressure monitoring and pulse oximetry [19–22, 24, 27, 30, 31, 33, 36, 37, 39, 44, 47, 48]. Documents referring to moderate sedation and documents that did not specify the level of sedation generally recommended electrocardiography only for select cases. These cases included patients with cardiac or pulmonary disease [22, 30, 33, 36, 39, 44], elderly patients [39], or prolonged procedures [39]. A guideline from Cancer Care Ontario (CCO) [39] also recommended the same factors to be considered when determining whether electrocardiography is needed for patients undergoing deep sedation. Five documents suggested routine use of electrocardiography for patients undergoing propofol sedation [19, 27, 29, 37, 47], whereas two documents recommended selective use, in particular for patients with cardiac [24, 48] and/or pulmonary disease [24]. Recommendations for capnography varied. For patients undergoing moderate sedation, a guideline from the ASGE [21] and a joint statement from the ASGE, American College of Gastroenterology (ACG), and AGA [42] both stated that there was insufficient data to recommend routine use of capnography. For patients undergoing deep sedation, two documents from the ASGE [21, 27] stated that capnography may be considered. For patients specifically undergoing sedation with propofol, there was no consensus on the use of capnography – use was recommended for all patients by the BSG [29], recommended to be considered by the ASGE [27], and recommended only in select cases by the ESGE [24]; statements that routine use is not supported were made by CAG [47] and ASGE [49]. Similarly, documents that did not specify the level of sedation also differed in recommendations for capnography, which was recommended for use by the CSGNA [44] and SSGE [31], while the GSGMD [36] and GESA [20] stated capnography may be considered. The AGA [22] and ESGE [24] did not recommend routine use of the bispectral index (BIS)/electroencephalography (EEG) during moderate sedation or NAAP, respectively; the GSGMD [36] stated that a benefit to EEG monitoring has not been demonstrated for sedated patients (no specific level of sedation identified).

**Table 4:** Summary of recommendations for equipment required to monitor sedated patients

	Moderate sedation	Deep sedation	Propofol	Sedation practices in general
*Non-Invasive Blood Pressure Monitoring*	ASGE [21] CCO [38, 39]	CCO [38, 39]	ESGE [24] DSRPGSA [19] ASGH [37] CAG [47] ASGE [27] ISDE	GSGMD [36] CSGNA [44] AGA [22] GESA [20] ASGE [27] SAGES [30]
*Pulse Oximetry*	ASGE [21] CCO [38, 39]	CCO [38, 39]	ESGE [24] DSRPGSA [19] CAG [47] ASGE [27] ISDE [48]	GSGMD [36] CSGNA [44] AGA [22] ASGH [37] SSGE [31, 33] GESA [20] SAGES [30]
*Capnography*	*Insufficient evidence:* ASGE [21] ASGE,ACG,AGA [42]	*Can be considered:* ASGE [21, 27]	*Recommended:* BSG [29] *Consider:* ASGE [27] *Select cases:* ESGE [24] ^[b]^ *Routine use not supported:* CAG [47] ASGE [49]	*Recommended:* CSGNA [44] SSGE [31] *Consider* GSGMD [36] GESA [20] *Insufficient evidence:* AGA [22]
*Electrocardiography*	*For select patients:* CCO [38, 39] ^[a]^	*For select patients:* CCO [38, 39] ^[a]^	BSG [29] DSRPGSA [19] ASGH [37] CAG [47] ASGE [27] *Select patients only:* ESGE [24] ^[c]^ ISDE [48] ^[d]^	*For select patients:* GSGMD [36] ^[e]^ CSGNA [44] ^[f]^ AGA [22] ^[g]^ SSGE [33] ^[h]^ GESA [20] ^[i]^ SAGES [30] ^[j]^
*Bispectral index monitoring*	*Not recommended:* AGA [22]	–	*Not recommended:* ESGE [24]	*Not recommended:* GSGMD [36]

*ACG* American College of Gastroenterology, *AGA* American Gastroenterological Association, *ASGE* American Society for Gastrointestinal Endoscopy, *ASGH* Austrian Society of Gastroenterology and Hepatology, *BSG* British Society of Gastroenterology, *CAG* Canadian Association of Gastroenterology, *CCO* Cancer Care Ontario, *CSGNA* Canadian Society of Gastroenterology Nurses and Associations, *DSRPGSA* Danish Secretariat for Reference Programmes for Gastroenterology, Surgery and Anaesthetics, *ESGE* European Society of Gastrointestinal Endoscopy, *GESA* Gastroenterological Society of Australia, *GSGMD* German Society for Gastroenterology, Digestive and Metabolic Diseases, *ISDE* Italian Society of Digestive Endoscopy, *SAGES* Society of American Gastrointestinal and Endoscopic Surgeons, *SSGE* Spanish Society of Gastrointestinal Endoscopy

^a^Reasonable for high-risk populations: history of cardiac or pulmonary disease, elderly patients, long procedures

^b^High-risk patients, intended deep sedation, long procedures

^c^History of cardiac and/or pulmonary disease

^d^Patients with specific cardiovascular risk

^e^Patients who have severe heart disease or expected arrhythmic problems

^f^If cardiac history may negatively impact outcomes

^g^High-risk patients (including those with a history of dysrhythmias)

^h^In patients with heart diseases

^i^According to the clinical status of the patient

^j^Patients with a history of cardiac disease

## Discussion

This review synthesized recommendations on sedation practice for routine GI endoscopy from 19 guidelines and 7 position statements. Overall, there was no consensus on optimal depth of sedation or sedative agents. While we found consistency in recommendations for the administration and monitoring of moderately sedated patients, documents varied considerably in their recommendations for the healthcare personnel capable of administering propofol and monitoring patients sedated with propofol, and the need for capnography during sedated GI endoscopy. Few documents provided a grade or level of evidence in support of their recommendations.

For patients undergoing routine GI endoscopy under moderate sedation, documents generally agree that sedation could be provided by a registered nurse, under the supervision of the physician, and that this nurse, in addition to monitoring the sedated patient, could perform brief, interruptible tasks to assist the endoscopist. Therefore, in the setting of moderate sedation, the presence of the endoscopist and a single, trained registered nurse was generally deemed sufficient. Appropriate training for an individual providing moderate sedation and monitoring patients sedated to this level includes an understanding of the pharmacology of the sedatives being used, which can be achieved through a course in sedation administration, and knowing how to respond to sedative-related complications, including rescuing patients who enter a deeper state of sedation. Certification in providing at least basic life support was also recommended.

Despite the increasing use of propofol for GI endoscopy [51, 52], we found wide variation in recommendations pertaining to most aspects of administering and monitoring patients sedated with propofol. For example, while some documents stated that it is not cost-effective for anesthesiologists to administer propofol in the setting of low-risk patients [31, 33, 48, 49], others advocated that propofol only be administered by anesthesia personnel or trained medical practitioners separate from the endoscopist [20, 29]. These differences likely reflect jurisdictional regulations and restrictions related to the administration of propofol by non-anesthesiologists; the evidentiary base for these recommendations was unclear. There was consistency, however, that when propofol is used for sedation in GI endoscopy, that monitoring the patient be the sole responsibility of an appropriately trained individual. This would require that at least 3 individuals be present for procedures performed with propofol: the endoscopist, an assistant, and an individual tasked with monitoring the patient. Additionally, specific training in the administration of propofol and skills in advanced airway management were recommended.

The inconsistency among the guidelines and position statements included in this review highlights the wide variability in sedation practices internationally. While certain jurisdictions routinely employ NAAP sedation, other regions require the presence of an anesthesiologist or trained anesthesia personnel when propofol is given [40]. This is important as differences in recommendations for the skills, training, and credentials of the individual responsible for monitoring a patient sedated with propofol could have serious safety and economic implications. Whereas recommendations for individuals monitoring moderately sedated patients included that they be capable of rescuing patients from deep sedation, recommendations for individuals monitoring patients sedated with propofol included that they be capable of rescuing patients from general anesthesia [27]. Given that endoscopy with propofol sedation is relatively common in certain jurisdictions, it is imperative that future work establish consensus on minimum requirements for the skills and training of individuals administering this agent and the equipment required for monitoring patients during both routine and complex GI endoscopy.

Our review used a systematic approach to identify recommendations from both guidelines and position statements, as both document types may contain recommendations that are currently guiding clinical care. We used a structured approach to assess the quality of included documents and the evidentiary base for recommendations. Additionally, we examined recommendations made in the setting of moderate and deep sedation separately, as well as those pertaining specifically to the use of propofol, and those that were not tied to a specific level of sedation. The results of our review can, therefore, be adapted to various clinical contexts based on individual endoscopists’ practices.

Our review is not without limitations. We used the AGREE II tool to assess the methodological rigor of documents and found that most documents scored poorly. For consistency, we applied the AGREE II tool to both guidelines and position statements; however, we acknowledge that position statements are unlikely to undergo the same development and reporting processes as guidelines and would advise caution when interpreting the scores of these statements. Additionally, in some documents, it was unclear whether certain statements were provided as recommendations or simply as a review of the literature; this uncertainty may have led us to misclassify some statements as recommendations. To mitigate against this risk, all recommendations were extracted by two reviewers and discrepancies were discussed.

## Conclusions

In conclusion, we found a lack of consensus as to the optimal level of sedation and agent to be used. The results of this review demonstrate consensus regarding the administration of moderate sedation and the monitoring of patients sedated to this level. However, documents varied in the recommended class of drugs for sedating patients undergoing GI endoscopy, the number and types of healthcare professionals that should be present during the procedure, particularly those performed with propofol, and the equipment needed to safely monitor these patients. Importantly, many of these differing recommendations were made without a sound evidentiary base. The lack of supporting evidence for the recommendations provided in the documents reviewed highlights the need for better evidence to guide GI endoscopy practices. Current variations in recommended practice are generally not evidence-based with potential implications for patient safety and procedural efficiency.

## Supplementary Information

**Additional file 1:** Appendices.

## Data Availability

Data extraction forms available from authors upon request.

## Abbreviations

ACG: American College of Gastroenterology
ACLS: Advanced Cardiac Life Support
AGREE: Appraisal of Guidelines for Research & Evaluation
ASA: American Society of Anesthesiologists
ASGE: American Society of Gastrointestinal Endoscopy
ASGH: Austrian Society of Gastroenterology and Hepatology
BIS: Bispectral Index
BSG: British Society of Gastroenterology
CAG: Canadian Association of Gastroenterology
CCO: Cancer Care Ontario
CSGNA: Canadian Society of Gastroenterology Nurses and Associates
DSRPGSA: Danish Secretariat for Reference Programmes for Gastroenterology, Surgery, and Anaesthetics
EC: European Commission
EEG: Electroencephalography
ERCP: Endoscopic Retrograde Cholangiopancreatography
ESGE: European Society of Gastrointestinal Endoscopy
ESGENA: European Society of Gastrointestinal Endoscopy Nurses and Associates
EUS: Endoscopic Ultrasound
FSDE: French Society of Digestive Endoscopy
GI: Gastrointestinal
GESA: Gastroenterological Society of Australia
GSGMD: German Society for Gastroenterology, Digestive and Metabolic Diseases
JAG: Joint Advisory Group on Gastrointestinal Endoscopy
NAAP: Non-Anesthesiologist Administered Propofol
PRISMA: Preferred Reporting Items for Systematic Review and Meta-Analysis
PRISMA-P: Preferred Reporting Items for Systematic Review and Meta-Analysis Protocols
SAGES: Society of American Gastrointestinal and Endoscopic
SGNA: Society of Gastroenterology Nurses and Associates
SSGE: Spanish Society of Gastrointestinal Endoscopy
