# Supplementary material for: Sedation practices for routine gastrointestinal endoscopy: a systematic review of recommendations
Source: BMC Gastroenterol. 2021 Jan 7;21:22. doi: 10.1186/s12876-020-01561-z (PMC7792218; doi:10.1186/s12876-020-01561-z)
Supplement: Supplementary file 1 — Additional file 1: Appendices. [file 12876_2020_1561_MOESM1_ESM.docx]

**Supplementary Materials**

**Supplemental Table 1.** Characteristics of included documents

**Supplemental Table 2.** Recommendations for individuals capable of administering sedation

**Supplemental Table 3.** Recommendations for individuals responsible for monitoring sedated patients

**Supplemental Table 4.** Recommendations for the skills and training required to administer sedation and monitor sedated patients

**Appendix 1.** Sources searched and search strategy

**Appendix 2.** Articles excluded through full-text screening

**Supplemental Table 1.** Characteristics of included documents

| **Document Developer** | **Year of Publication** | **Country of Publication** | **Procedure(s) Covered** |
| --- | --- | --- | --- |
| ***Guidelines (n=19)*** | | | |
| American Society for Gastrointestinal Endoscopy^1^ | 2006 | U.S. | GI endoscopy |
| British Society of Gastroenterology^2^ | 2006 | Britain | GI endoscopy |
| French Society of Digestive Endoscopy^3^ | 2006 | France | Colonoscopy |
| American Gastroenterological Association^4^ | 2007 | U.S. | GI endoscopy |
| Austrian Society of Gastroenterology and Hepatology^5^ | 2007 | Austria | GI endoscopy |
| Society of American Gastrointestinal and Endoscopic Surgeons^6^ | 2009 | U.S. | GI endoscopy |
| American Society for Gastrointestinal Endoscopy^7^ | 2010 | U.S. | GI endoscopy |
| Danish Secretariat for Reference Programmes for Gastroenterology, Surgery and Anaesthetics^8^ | 2011 | Denmark | GI endoscopy |
| Spanish Society of Gastrointestinal Endoscopy^9^ | 2012 | Spain | Colonoscopy |
| European Commission^10^ | 2012 | Europe | Colonoscopy |
| American Society of Gastrointestinal Endoscopy^11^ | 2013 | U.S. | GI endoscopy |
| Gastroenterological Society of Australia^12^ | 2014 | Australia | GI endoscopy |
| American Society for Gastrointestinal Endoscopy^13^ | 2014 | U.S. | GI endoscopy |
| Cancer Care Ontario^14,15^ | 2014 | Canada | Colonoscopy |
| Spanish Society of Gastrointestinal Endoscopy^16^ | 2014 | Spain | GI endoscopy |
| European Society of Gastrointestinal Endoscopy & European Society of Gastrointestinal Endoscopy Nurses and Associates^17^ | 2015 | Europe | GI endoscopy |
| German Society for Gastroenterology, Digestive and Metabolic Diseases^18^ | 2016 | Germany | GI endoscopy |
| American Society for Gastrointestinal Endoscopy^19^ | 2018 | U.S. | GI endoscopy |
| Joint Advisory Group on Gastrointestinal Endoscopy^20^ | 2018 | Britain | GI endoscopy |
| ***Position Statements (n=7)*** | | | |
| Canadian Association of Gastroenterology^21^ | 2008 | Canada | GI endoscopy |
| Society of Gastroenterology Nurses and Associates^22^ | 2008 | U.S. | GI endoscopy |
| American Society for Gastrointestinal Endoscopy^23^ | 2009 | U.S. | GI endoscopy |
| American Society for Gastrointestinal Endoscopy, American College of Gastroenterology & American Gastroenterological Association^24^ | 2012 | U.S. | Colonoscopy & Gastroscopy |
| Canadian Society of Gastroenterology Nurses and Associates^25^ | 2016 | Canada | GI endoscopy |
| Society of Gastroenterology Nurses and Associates^26^ | 2016 | U.S. | GI endoscopy |
| Italian Society of Digestive Endoscopy^27^ | 2017 | Italy | GI endoscopy |

GI = Gastrointestinal; U.S = United States

**Supplemental Table 2.** Recommendations for individuals capable of administering sedation

| **Subject** | | | **Document** | **Recommendation of Statement (Quote)** | **Strength** | **Level of evidence** |
| --- | --- | --- | --- | --- | --- | --- |
| *Moderate sedation* | | | | | | |
|  | Can be administered by a nurse who is directed by a physician | | ASGE^13^ | Sedation should be directed by the physician who is credentialed and privileged to do so and can be administerd by an RN | - | - |
|  |  |  | ASGE^7^ | It is recommended that for endoscopy with moderate sedation, a single RN may administer sedation under physician supervision | - | - |
|  |  |  | ASGE^19^ | Medications targeting minimal and moderate sedation generally can be administered in an incremental fashion by an appropriately trained RN under the supervision of an endoscopist | - | - |
|  |  |  | SGNA^22^ | Registered nurses trained and experienced in gastroenterology nursing and endoscopy can administer and maintain moderate sedation and analgesia by the order of a physician | - | - |
|  | Should be administered by a physician other than the endoscopist | | GESA^12^ | In situations other than those when an anesthetist must be present, administration of sedation and/or analgesia and monitoring of the patient should be performed by another appropriately trained (i.e. advanced life support skills) medical or dental practitioner working with the proceduralist  Intravenous anaesthetic agents may only be used by a medical or dental practitioner trained in their use | - | - |
| *Deep sedation* | | | | | | |
|  | Should be administered by an anesthesia professional | | ASGE^13^ | Most institutions require that deep sedation be administered by an anesthesia professional such as an Anesthesiologist, Certified Registered Nurse Anesthetist (CRNA), or Anesthesiologist Assistant who are credentialed and privileged to do so. In this situation, the anesthesia provider should be responsible for administering sedation and monitoring the patient | - | - |
|  |  |  | GESA^12^ | Techniques intended to produce deep sedation or general anesthesia must not be used unless there is present an anesthetist or other appropriately trained and credentialed medical specialist within his/her scope of practice | - | - |
|  |  |  | SGNA^22^ | The SGNA supports the position of the ASGE that the assistance of anesthesiologists should be considered in patients undergoing prolonged procedures, requiring deep sedation |  |  |
| *Propofol* | | | | | | |
|  | Should not be administered by nurses | | CSGNA^25^ | It is currently not within the scope of practice for an endoscopy nurse to administer propofol | - | - |
|  |  |  | GESA^12^ | Intravenous anesthetic agents such as propofol must only be used by a second medical or dental practitioner trained in their use because of the risk of unintentional loss of consciousness. These agents must not be administered by the proceduralist | - | - |
|  |  |  | BSG^2^ | Patients requiring anaesthetic support in the Gastrointestinal Endoscopy Unit: …Any patient being sedated with IV propofol | - | - |
|  | Non-anesthesiologist propofol administration can be considered | | GSGMD^18^ | Propofol administration during the procedure by a properly trained and experienced person [non-physician], who has this as his or her sole task on a physician’s instruction, may be considered | Statement | 1 |
|  |  |  | DSRPGSA^8^ | A nurse, under the responsibility of the doctor (non-anesthetist) responsible for the examination, is responsible for planning and performing NAPS based on the patient’s age, underlying health status, and the nature of the procedure | - | - |
|  |  |  | AGA^4^ | Gastroenterologist-directed administration of propofol is a safe and effective alternative to sedation with opioids and benzodiazepines | - | - |
|  |  |  | SSGE^9^ | Safety of colonoscopy with propofol sedation when administered by a non-anesthesiologist is high and similar to the risk of sedation with benzodiazepines, regarding to hypoxia, hypotension, and bradycardia | A | 1a |
|  |  |  | SSGE^16^ | The use of propofol by endoscopists or trained nurses is as safe as traditional sedatives when monitoring is adequate | A | 1++ |
|  |  |  |  | Sedation with propofol administered by non-anesthetist clinicians may improve endoscopy unit efficiency | A | 1+ |
|  |  |  | CAG^21^ | Propofol is safe for use as a conscious sedation agent for endoscopy, when used by appropriately trained endoscopists and/or endoscopy nurses  Appropriately trained endoscopy nurses can also administer propofol for endoscopy  For low-risk endoscopy patients, routine involvement of anesthesiologists to administer propofol is not required | - | - |
|  |  |  | ASGE^23^ | NAAP sedation improves practice efficiency when compared to standard sedation  The use of anesthesiologist-administred sedation for healthy, low-risk patients undergoing routine GI endoscopy results in higher costs with no proven benefit with respect to patient safety or procedural efficiency | C | 2 |
|  |  |  | ISDE^27^ | NAAP is safe when administered by trained personnel in carefully selected patients | - | - |
|  | An anesthesiologist should be readily available when non-anesthesiologist propofol sedation is used | | DSRPGSA^8^ | NAPS may only be performed if there is an anesthetist in the immediate vicinity | - | - |
|  |  |  | SSGE^16^ | In endoscopy units where deep sedation is used an anaesthesiologist or intensivist should be available within 5 minutes | C | 2+ |
|  | Patient and procedure factors to consider when determining whether an anesthesiologist is required | |  |  |  |  |
|  |  | ASA class | ESGE^17^ | We suggest primary involvement of an anesthesiologist in patients with ASA class ≥3 | D | 4 |
|  |  |  | GSGMD^18^ | NAPS should not be done for patients with an ASA grade IV-V | - | - |
|  |  |  | DSRPGSA^8^ | Propofol sedation by non-anaesthetics may only be performed on patients in ASA classes 1 and 2…Patients in ASA group 3 and above, and children under 10, may only be sedated with the assistance of staff trained in anaesthetics | - | - |
|  |  |  | SSGE^9^ | Assistance from an anesthesiologist when using propofol in healthy people (ASA I-II) is very expensive and it has not shown any improvement in the patient safety or in the procedure outcome | - | 2c |
|  |  |  | SSGE^16^ | Deep sedation with propofol for basic endoscopic procedures and patients with ASA I-II risk may be carried out effectively and safely in the absence of dedicated sedation staff | C | 2+ |
|  |  |  |  | For procedures performed in patients with advanced ASA scores (>III)…the presence of an anesthesiologist or intensivist is to be recommended | D | 4 |
|  |  |  | CAG^21^ | Anesthesiology support should be considered for: ASA class III or higher | - | - |
|  |  |  | ISDE^27^ | Anesthesiologist support is required for ASA IV and ASA V cases  Anesthesiologist support should always be considered for:…ASA III cases | - | - |
|  |  | Mallampati class or facial features | ESGE^17^ | We suggest primary involvement of an anesthesiologist in patients…with a Mallampati’s class ≥3  The presence of some patient-related risk factors may trigger consideration of primary involvement of an anesthesiologist during endoscopy. Such factors are, for example: …patients with dysmorphic facial features or oral abnormalities, such as a small opening (<3cm in an adult), high arched palate, or macroglossia…patients with jaw abnormalities, such as micrognathia | D | 4 |
|  |  | Other factors suggestive of difficult intubation or ventilation | SSGE^16^ | For procedures performed in patients with…short neck, sleep apnea…the presence of an anesthesiologist or intensivist is to be recommended | D | 4 |
|  |  |  | ESGE^17^ | We suggest primary involvement of an anesthesiologist in patients with…other conditions that put them at risk of airway obstruction (e.g. pharyngolaryngeal tumors) | D | 4 |
|  |  |  |  | The presence of some patient-related risk factors may trigger consideration of primary involvement of an anesthesiologist during endoscopy. Such factors are, for example: a history of stridor, snoring, or OSA;…patients with neck or cervical spine abnormalities, tracheal deviation, or advanced rheumatoid arthritis | - | - |
|  |  |  | DSRPGSA^8^ | The following patients are unsuitable for propofol sedation by non-anaesthetic personnel due to increased risk of airway complications: BMI ≥35, non-compliance with fasting guidelines, high respiratory assessment score, equal to or above 4, risk of gastric retention | - | - |
|  |  |  | CAG^21^ | Anesthesiology support should be considered for: individuals with difficult anatomy for ventilatory support (e.g. obesity or thick necks) | - | - |
|  |  |  | ISDE^27^ | Anaesthesiologist support should always be considered for: patients with anatomic obstacles for ventilatory support (e.g. obesity, thick necks) | - | - |
|  |  | Patients with other high risk conditions | DSPRGSA^8^ | The following patients are unsuitable for propofol sedation by non-anaesthetic personnel due to increased risk of airway complications: …acute upper GI hemorrhage, sub-acute bowel obstruction/ileus, achalasia, sleep apnea  Patients with low oxygen saturation, i.e. below 95% despite use of supplemental oxygen, may only be sedated with the assistance of an anaesthetist | - | - |
|  |  |  | SSGE^16^ | For procedures performed in patients with…chronic decompensated serious diseases…the presence of an anesthesiologist or intensivist is to be recommended | D | 4 |
|  |  | Long or complex procedures | ESGE^17^ | We suggest primary involvement of an anesthesiologist…in cases where a long-lasting procedure is anticipated | D | 4 |
|  |  |  | DSRPGSA^8^ | Propofol sedation by non-anaesthetists may only be performed…in examinations expected to last less than one hour | - | - |
|  |  |  | CAG^21^ | Anesthesiology support should be considered for: prolonged or high-risk interventional procedures | - | - |
|  |  |  | SSGE^16^ | For complex therapeutic procedures, having an additional, qualified person responsible for sedation is advisable | D | 4 |
|  |  |  | ISDE^27^ | Anesthesiologist support should always be considered for: …long lasting or high risk interventional procedures | - | - |
|  |  | Other risk factors | ESGE^17^ | We suggest primary involvement of an anesthesiologist in patients…who chronically receive significant amounts of narcotic analgesics | Weak | Low |
|  |  |  |  | The presence of some patient-related risk factors may trigger consideration of primary involvement of an anesthesiologist during endoscopy. Such factors are, for example: …patients receiving significant amounts of narcotic analgesics chronically or who for other reasons may be tolerant to agents used during sedation and analgesia | - | - |
|  |  |  |  | If a patient proves difficult to sedate adequately for the examination purpose, endoscopy termination and referral to an anesthesiologist should be considered | D | 4 |
|  |  |  | DSRPGSA^8^ | The following patients are unsuitable for propofol sedation by non-anaesthetic personnel due to increased risk of airway complications: …previous problems with anesthesia | - | - |
|  |  |  | ISDE^27^ | Anaesthesiologist support should always be considered for:…uncooperative patients | - | - |
| *Sedation practice in general* | | | | | | |
|  | The role of nurses in the administration of sedation | | CSGNA^25^ | Competent Registered Nurses training in the field of gastroenterology, procedural sedation, medication management, and airway management may be given responsibility of administering procedural sedation under direct order and supervision of the physician | - | - |
|  |  |  | ASGH^5^ | There must always be an individual present who is responsible for the administration of sedatives or anesthetics. Depending on the degree of sedation on the one hand and on the presence of risk factors that could lead to a requirement for intubation on the other, this individual can be a specially trained assistant or nurse, a member of the general medical staff, or an anesthesiologist | - | - |
|  |  |  | ASGE^13^ | LPNs and UAPs are not qualified to administer sedation | - | - |
|  |  |  | GESA^12^ | This person [assistant to proceduralist who is appropriately trained in observation and monitoring of sedated patients] may, if appropriately trained, administer sedative and/or analgesic drugs under the direct supervision of the proceduralist | - | - |
|  |  |  | SAGES^6^ | Registered professional nurses (RNs) who administer analgesic or sedative drugs as part of a medical procedure (including but not limited to certified RN anesthetists)…must function in accordance with their scope of practice | - | - |
|  | Intravenous sedation should be administered by an anesthesiologist | | FSDE^3^ | Intravenous sedation by a doctor who is not an anaesthetist is not recommended outside of clinical trials | - | - |
|  | Patient and procedure factors to consider when determining whether an anesthesiologist is required | |  |  |  |  |
|  |  | ASA class | GSGMD^18^ | We recommend that calling in an anesthesiologist should be considered for patients with a high risk profile. These include: high ASA grade (III-IV) | - | - |
|  |  |  | AGA^4^ | The use of an anesthesia professional should be strongly considered for ASA physical status IV and V patients | - | - |
|  |  |  | GESA^12^ | …in such cases, an anesthetist should be present for the care of the patient. These patients include…patients in ASA grades 4-5 | - | - |
|  |  |  | ASGE^19^ | Anesthesia provider assistance should be considered in the following situations: …increased risk for adverse event because of severe comorbidity (ASA class IV or V) | - | - |
|  |  |  | SSGE^16^ | Recommendations for anesthetic care during GI endoscopy: 3. Increased risk of complications because of severe comorbidity (ASA 4 or higher) | - | - |
|  |  | Mallampati class or facial features | GSGMD^18^ | We recommend that calling in an anesthesiologist should be considered for patients with a high risk profile. These include: …severely restricted mouth opening <2cm; Mallampati grade 3 or 4; or a restricted hyoid-to-chin distance <4cm | - | - |
|  |  |  | SSGE^16^ | Recommendations for anesthetic care during GI endoscopy: 7. Evidence of dysmorphic face (Trisomy 21, Pierre-Robin sequence); 8. Mouth abnormalities (e.g. mouth opening <3cm, protruding incisors, macroglossia, gothic plate, tonsillar hypertrophy, Mallampati scale = 4)…10. Mandible abnormalities (retrognathia, micrognathia, trismus, severe dental malocclusion | - | - |
|  |  |  | AGA^4^ | Other possible indications for an anesthesia specialist include patients with…morbid obesity | - | - |
|  |  | Other factors suggestive of difficult intubation or ventilation | GSGMD^18^ | We recommend that calling in an anesthesiologist should be considered for patients with a high risk profile. These include: …the presence of pathological anatomical features associated with a higher risk of airway obstruction during the intervention (e.g. craniofacial malformation; lingual, laryngeal, or hypopharyngeal tumor; severely restricted mobility of the cervical spine | - | - |
|  |  |  | GESA^12^ | …in such cases, an anesthetist should be present for the care of the patient. These patients include…morbid obesity, significant obstructive sleep apnea, or known or suspected difficult endotracheal intubation…the potential for aspiration of stomach contents (which may necessitate endotracheal intubation) | - | - |
|  |  |  | ASGE^19^ | Anesthesia provider assistance should be considered in the following situations: …increased risk for airway obstruction because of anatomic variant | - | - |
|  |  |  | SSGE^16^ | Recommendations for anesthetic care during GI endoscopy: 5. Prior history of laryngeal stridor; 6) history of sleep apnea…9) Neck abnormalities (decreased hyoid-chin distance, short thick neck, limited cervical extension, cervical spine conditions or trauma, severe tracheal deviation | - | - |
|  |  | Patients with other high risk conditions | GESA^12^ | …in such cases, an anesthetist should be present for the care of the patient. These patients include the elderly, those with severely limiting heart, cerebrovascular, lung, liver, or renal disease…acute GI bleeding particularly with cardiovascular compromise or shock, severe anemia | - | - |
|  |  |  | ASGE^19^ | We recommend anesthesia provider-administered sedation be considered for…patients with multiple medical comorbidities or at risk for airway compromise | - | Moderate |
|  |  |  | BSG^2^ | Patients requiring anesthetic support in the GI endoscopy unit (elective cases): …outflow obstruction and any serious form of cardiac or pulmonary compromise | - | - |
|  |  | Long or complex procedures | GSGMD^18^ | We recommend that calling in an anesthesiologist should be considered for patients with a high risk profile. These include: …difficult endoscopic intervention | - | - |
|  |  |  | AGA^4^ | Endoscopic procedures that may require an anesthesia specialist include ERCP, stent placement in the upper GI tract, EUS, and complex therapeutic procedures (e.g. ESD, plication of the cardioesophageal junction, EGD with drainage of pseudocyst) | - | - |
|  |  |  | ASGE^19^ | We recommend anesthesia provider-administered sedation be considered for complex endoscopic procedures | - | Moderate |
|  |  |  | SSGE^16^ | Recommendations for anesthetic care during GI endoscopy: 1. Endoscopic procedures that are urgent, prolonged, or therapeutically complex subject to deep sedation or GA | - | - |
|  |  | Other risk factors | AGA^4^ | Other possible indications for an anesthesia specialist include patients with a history of alcohol or substance abuse, pregnancy, morbid obesity, neurologic or neuromuscular disorders, and patients who are uncooperative or delirious | - | - |
|  |  |  | GESA^12^ | …in such cases, an anesthetist should be present for the care of the patient. These patients include…previous adverse events due to sedation, analgesia, or anesthesia | - | - |
|  |  |  | ASGE^19^ | Anesthesia provider assistance should be considered in the following situations: …anticipated intolerance to standard sedatives | - | - |
|  |  |  | SSGE^16^ | Recommendations for anesthetic care during GI endoscopy: 2. Intolerance, paradoxical reactions or allergy to standard sedation schedules | - | - |
|  |  |  | BSG^2^ | Patients requiring anesthetic support in the GI endoscopy unit (elective cases): patients with severe learning difficulties; patients in whom sedation has previously failed (or is likely to fail) e.g. certain alcoholic or drug addicted patients who may prove difficult to sedate and/or have poor venous access; phobic or uncooperative patients who insist on being ‘put to sleep’ | - | - |

ACG = American College of Gastroenterology; AGA = American Gastroenterological Association; ASGE = American Society for Gastrointestinal Endoscopy; ASGH = Austrian Society of Gastroenterology and Hepatology; BSG = British Society of Gastroenterology; CAG = Canadian Association of Gastroenterology; CCO = Cancer Care Ontario; CSGNA = Canadian Society of Gastroenterology Nurses and Associations; DSRPGSA = Danish Secretariat for Reference Programmes for Gastroenterology, Surgery and Anaesthetics; EC = European Commission; ESGE = European Society of Gastrointestinal Endoscopy; ESGENA = European Society of Gastrointestinal Endoscopy Nurses and Associates; FSDE = French Society of Digestive Endoscopy; GESA = Gastroenterological Society of Australia; GSGDMD = German Society for Gastroenterology, Digestive and Metabolic Diseases; ISDE = Italian Society of Digestive Endoscopy; JAG = Joint Advisory Group; SAGES = Society of American Gastrointestinal and Endoscopic Surgeons; SGNA = Society of Gastroenterology Nurses and Associates; SSGE = Spanish Society of Gastrointestinal Endoscopy

**Supplemental Table 3.** Recommendations for individuals responsible for monitoring sedated patients

| **Subject** | | **Document** | **Recommendation or Statement (Quote)** | **Strength** | **Level of Evidence** |
| --- | --- | --- | --- | --- | --- |
| *Moderate sedation* | | | | | |
|  | There should be two individuals in the endoscopy suite during moderate sedation | CSGNA^25^ | If a moderate sedation level is achieved for the procedure, two health professionals are recommended in the endoscopy suite | - | - |
|  | A single nurse can monitor a moderately sedated patient and perform brief, interruptible tasks | AGA^4^ | During moderate sedation, the person assigned responsibility for patient assessment may also perform tasks that are interruptible and of short duration | - | - |
|  |  | ASGE^13^ | When moderate sedation is the target, a nurse should monitor the patient and can perform interruptible tasks | - | - |
|  |  | ASGE^7^ | It is recommended that for endoscopy with moderate sedation, a single RN may administer sedation under physician supervision and assist with the technical portion of the endoscopic procedure, provided that these tasks be interruptible | - | - |
|  |  | ASGE^19^ | Patient response to administered sedatives and analgesics should be monitored by a nurse (generally an RN), whose primary responsibility is patient monitoring. In this setting, the RN can perform short, interruptible tasks in addition to monitoring the patient | - | - |
|  |  | SGNA^22^ | The RN is responsible for monitoring and assessing the patient receiving moderate sedation and analgesia throughout the diagnostic and therapeutic endoscopic procedure  During moderate sedation, the registered nurse monitoring the patient may assist with minor, interruptible tasks once the patient’s level of sedation/analgesia and vital signs have stabilized | - | - |
|  |  | SAGES^6^ | The individual administering conscious sedation or monitoring the patient cannot be involved in uninterruptible duties | - | - |
|  | Complex procedures or patients require a second assistant | ASGE^13^ | If more technical assistance is required, a second assistant (nurse, licensed practical nurse, or unlicensed assistive personnel) should be available to join the care team | - | - |
|  |  | ASGE^7^ | In patients who require more intensive or prolonged endoscopic interventions (e.g. difficult polypectomy, EUS, ERCP), a second assistant, who may be a UAP, LPN, or RN, should assist the procedure to allow the RN administering moderate sedation to remain focused on patient monitoring rather than on technical assistance | - | - |
|  |  | SGNA^22^ | Because of the importance assigned to managing the patient who is receiving sedation and analgesia, a second nurse or associate is required to assist the physician with those procedures that are complicated by the severity of the patient’s illness and/or the complex technical requirements associated with advanced diagnostic and therapeutic procedures | - | - |
| *Deep sedation* | | | | | |
|  | Requires an individual dedicated to monitoring the patient who has no other tasks | AGA^4^ | When deep sedation is planned, this individual should be dedicated to observation and monitoring and have no other procedure-related responsibilities | - | - |
|  |  | ASGE^13^ | The individual responsible for patient monitoring must be dedicated solely to that task and may not perform any other function during the procedure  A second staff person (RN, LPN, UAP) is required to assist with technical aspects of the procedure | - | - |
|  |  | ASGE^7^ | It is recommended that if an RN administers deep sedation under physician supervision, then staffing should include a second endoscopy assistant. This individual may be a UAP, LPN, or RN | - | - |
|  |  | SGNA^22^ | The SGNA recommends that the registered nurse be present to monitor the patient throughout procedures performed with deep sedation/analgesia. During deep sedation, the registered nurse should have no other responsibilities. Because of the importance assigned to managing the patient who is receiving sedation and analgesia, a second nurse or assistant is required to assist the physician | - | - |
|  | An anesthesia professional should monitor the patient | ASGH^5^ | When patients with known risk factors undergo endoscopy or endoscopic interventions under deep sedation who might require endotracheal intubation, the presence of an anesthesiologist might be desirable, depending on the intubation experience of the endoscopy team | - | - |
|  |  | ASGE^13^ | Most institutions require that deep sedation be administered by an anesthesia professional such as an Anesthesiologist, Certified Registered Nurse Anesthetist, or Anesthesiologist Assistant who are credentialed and privileged to do so. In this situation, the anesthesia provider should be responsible for administering sedation and monitoring the patient | - | - |
| *General Anesthesia* | | | | | |
|  | Requires an anesthesiologist | JAG^20^ | Some patients will require a general anesthetic; if this is the case then the anesthetist is responsible for monitoring the patient and advising on postanesthetic care | - | - |
|  | Requires an anesthesiologist and an individual to assist the anesthesiologist | GESA^12^ | If general anesthesia is intended, and especially in emergency situations where endotracheal intubation is planned, a fourth person to specifically assist the anesthetist throughout the procedure is required | - | - |
| *Propofol* | | | | | |
|  | Requires an individual dedicated to monitoring the patient who has no other tasks | ESGE^17^ | It is recommended that patients be continuously monitored by a person dedicated to NAAP | A | - |
|  |  | GSGMD^18^ | Subsequent monitoring by an experienced person with appropriate training (physician, nurse/physician assistant) may be considered | Statement | 1b |
|  |  |  | We recommend that the person responsible for monitoring must not have any other tasks during this time | A | 5 |
|  |  |  | Propofol administration during the procedure by a properly trained and experienced person, who has this as his or her sole task on a physician’s instruction, may be considered | Statement | 1 |
|  |  | DSRPGSA^8^ | In addition to the NAPS nurse, there must be a doctor and an assisting nurse in the examination room prior to initiation of propofol sedation.  The NAPS nurse is solely responsible for NAPS (observation and sedation of the patient) and does not participate in other tasks during the examination  The depth of the patient’s sedation and respiration are monitored continuously by the NAPS nurse | - | - |
|  |  | CAG^21^ | If the gastroenterologist administers the propofol, a nurse who cares only for the cardiorespiratory status of the patient should be present  Explicitly interpreted, two nurses (or one nurse and one endoscopy technician) would be required for cases involving propofol | - | - |
|  |  | ASGE^19^ | Trained personnel dedicated to the uninterrupted monitoring of the patient’s clinical and physiologic parameters throughout the procedure should be available | - | - |
|  |  | ISDE^27^ | Monitoring by a dedicated person is safer  Overall, it is generally recommended that nurses administering propofol must be dedicated to monitoring the patient and not involved in the endoscopic procedure | - | - |
|  | Whether an individual dedicated to monitoring the patient sedated with propofol depends on the complexity of the patient and procedure | SSGE^16^ | Deep sedation with propofol for basic endoscopic procedures and patients with ASA I-II risk may be carried out effectively and safely in the absence of dedicated sedation staff with no increase in the number of people inside the room | C | 2+ |
|  |  |  | For complex therapeutic procedures, having an additional, qualified person responsible for sedation is advisable | D | 4 |
|  |  |  | For procedures performed in patients with advanced ASA scores (>III) or with risk factors for sedation (short neck, sleep apnea, chronic decompensated serious diseases, etc.) the presence of an anesthesiologist or intensivist is to be recommended | D | 4 |
|  | A physician must be present throughout propofol sedation | DSRPGSA^8^ | A minimum of two people (one doctor and one nurse) must be present until the patient has woken up after sedation | - | - |
|  |  | ASGE^19^ | A physician should be present throughout propofol sedation and must remain immediately available until the patient meets discharge criteria | - | - |
| *Sedation practices in general* | | | | | |
|  | A minimum of one nurse is required | SGNA^26^ | All endoscopy settings must have a gastroenterology RN present during the pre-procedure phase of care, the procedure, and the post-procedure phase of care | - | - |
|  |  | AGA^4^ | A nurse or assistant with appropriate training in endoscopic sedation should be present throughout the endoscopic procedure | - | - |
|  | Sedation for endoscopy requires an individual dedicated to administering sedation and monitoring the sedated patient | GSGMD^18^ | For every endoscopy under sedation we recommend that one person should be solely responsible for the performance and monitoring of the sedation | A | 5 |
|  |  | GESA^12^ | Except for very light conscious sedation and/or analgesic techniques such as inhaled nitrous oxide or low dose oral sedation, there must be a minimum of three appropriately trained staff present: the proceduralist, the medical or dental practitioner administering the sedation and monitoring the patient, and at least one additional staff member to provide assistance to the proceduralist and/or the practitioner providing sedation as required | - | - |
|  | Nurses monitoring sedated patients should only assist with brief, interruptible tasks | SGNA^26^ | RNs administering procedural sedation must not have other responsibilities that would compromise their ability to adequately monitor the patient before, during, and after the procedure | - | - |
|  | An increase in staff is required for complex patients or procedures | GSGMD^18^ | We suggest that in all cases of an increased risk for the patient, or when a long, complex intervention is expected, a second physician (qualified in resuscitation and intensive care) should be present whose only task is the sedation and monitoring of the patient | B | 5 |
|  |  | SGNA^26^ | Additional personnel may be needed in some or all phases of procedure-related care in order to provide an efficient, cost-effective, and safe patient experience. The level of additional personnel is dictated by:   - Technical aspects and complexity of particular procedures (e.g. ERCP, PEG/PEJ, EUS, large polyp removal) - Acuity of the expected patient population - Types of anesthesia or level of sedation | - | - |
|  |  | CSGNA^25^ | If the procedure is deemed therapeutic, then a second Registered Nurse/Licensed Practical Nurse/Registered Practical Nurse is required to maintain patient safety. The Registered Nurse responsible for the airway and monitoring of the patient cannot also be the therapeutic equipment nurse (RN/LPN/RPN) | - | - |
|  |  | SAGES^6^ | During particularly complex or instrument-intensive procedures, in which the assistant is likely to be too busy assisting the physician to monitor the patient adequately, a second assistant must be made available to monitor and care for the patient | - | - |
|  |  | ASGH^5^ | There must always be an individual present who is responsible for the administration of sedatives or anesthetics. Depending on the degree of sedation on the one hand and on the presence of risk factors that could lead to a requirement for intubation on the other, this individual can be a specially trained assistant or nurse, a member of the general medical staff, or an anesthesiologist. This individual is responsible for monitoring during the endoscopic procedure | - | - |
|  | Role of the nurse when an anesthesia professional is present | SGNA^26^ | When an anesthesia provider is administering sedation, the RN will remain to provide continuity of care and assist the healthcare team | - | - |
|  |  | CSGNA^25^ | In situations where anaesthesia is involved in administering procedural sedation and monitoring of the patient, only one nurse is required in the procedure room to assist the endoscopist with therapeutics and can provide support to anaesthesia to maintain continuity of care | - | - |
|  |  | ASGE^13^ | When sedation and monitoring is provided by anesthesia personnel, a single additional staff person (nurse, licensed practical nurse, or unlicensed assistive personnel) is sufficient to assist with technical aspects of the procedure | - | - |
|  |  | GESA^12^ | The assistant to the medical or dental practitioner administering sedation/anesthesia must be exclusively available to that practitioner at induction of and emergence from sedation/anesthesia, and during the procedure as required | - | - |
|  |  | ASGE^7^ | In the setting of sedation administered by an anesthesia provider (ie anesthesiologist or certified RN anesthetist), it is recommended that staffing include an individual dedicated to endoscopic technical assistance. This individual may be a UAP, LPN, or RN | - | - |

**Supplemental Table 4.** Recommendations for the skills and training required to administer sedation and monitor sedated patients

| **Subject** | | **Document** | **Quote** | **Strength** | **Level of Evidence** |
| --- | --- | --- | --- | --- | --- |
| *Moderate Sedation* | | | | | |
|  | Formal training | CSGNA^25^ | To establish competency in this skill a Registered Nurse must be provided by their endoscopy department or institution an education module on procedural sedation, and pass examination of a written and practical component | - | - |
|  | Ability to rescue a patient who enters deep sedation | AGA^4^ | Physicians targeting moderate sedation (either with an opioid/benzodiazepine combination or propofol) should be capable of rescuing a patient who enters deep sedation | - | - |
|  |  | ASGE^1^ | For moderate (conscious) sedation, the endoscopist must have the skills to rescue a patient from deep sedation. These skills are similar to those taught in Basic Life Support (BLS) but also include the use of reversal agents | - | - |
|  |  | CSGNA^25^ | The RN must be knowledgeable of sedation overshoot and have the skill to correct or reverse the overshoot | - | - |
|  | Knowledge of the medications used | SGNA^22^ | The gastroenterology-registered nurse has education and experience in endoscopy, knowledge of medications used… | - | - |
|  |  | CSGNA^25^ | The formal training must include:   - Safe administration of sedative and analgesic medications used to establish a moderate level of sedation - Understanding of all sedation medications, pharmacology, side effects, and administration guidelines - Understanding of indications and contraindications of moderate sedation - Use of reversal agents for opioids and benzodiazepines | - | - |
|  | Ability to assess for and intervene if complications occur | SGNA^22^ | The gastroenterology-registered nurse has education and experience in endoscopy, knowledge of medications used, and the skills to assess, diagnose, and intervene in the event of complications | - | - |
|  |  | CSGNA^25^ | The Registered Nurse must be knowledgeable and skilled in assessing, diagnosing, and intervening in the event of complications | - | - |
|  | Skills in airway management | CSGNA^25^ | The formal training must include:   - ……Understanding airway management, assessment, and intervention | - | - |
|  | Basic life support certification | CSGNA^25^ | The RN must have basic cardiac life support (in hospitals) and ACLS (in private endoscopy clinics) | - | - |
|  | ACLS training may or may not be required for nurses | CSGNA^25^ | ACLS training [for nurses] is not required in institutions that have code response teams. In institutions where no code team exists then staff must be trained in ACLS  The RN must have basic cardiac life support (in hospitals) and ACLS (in private endoscopy clinics) | - | - |
| *Deep Sedation* | | | | | |
|  | Knowledge of medications used | SGNA^22^ | The SGNA recommend that registered nurses and physicians involved in the administration of deep sedation have additional training with emphasis on advanced airway management and treatment of cardiorespiratory complications. This may include, but is not limited to, advanced cardiac life support, pediatric advanced life support, additional advanced airway management training, and advanced training on medications that can be used to achieve deep sedation or can lead to or easily induce a state of general anesthesia | - | - |
|  | Ability to rescue the patient from general anesthesia | ASGE^1^ | For deep sedation (including all uses of propofol), the endoscopist must have the ability to rescue the patient from general anesthesia, including managing a compromised airway | - | - |
|  | Advanced airway management and ability to manage cardiorespiratory complications | AGA^4^ | Physicians targeting deep sedation require additional training with emphasis on advanced airway management and treatment of cardiorespiratory complications | - | - |
|  |  | SGNA^22^ | The SGNA recommend that registered nurses and physicians involved in the administration of deep sedation have additional training with emphasis on advanced airway management and treatment of cardiorespiratory complications. This may include, but is not limited to, advanced cardiac life support, pediatric advanced life support, additional advanced airway management training, and advanced training on medications that can be used to achieve deep sedation or can lead to or easily induce a state of general anesthesia | - | - |
|  |  | ASGE^1^ | For deep sedation (including all uses of propofol), the endoscopist must have the ability to rescue the patient from general anesthesia, including managing a compromised airway | - | - |
|  | Advanced cardiac life support training | SGNA^22^ | The SGNA recommend that registered nurses and physicians involved in the administration of deep sedation have additional training with emphasis on advanced airway management and treatment of cardiorespiratory complications. This may include, but is not limited to, advanced cardiac life support, pediatric advanced life support, additional advanced airway management training, and advanced training on medications that can be used to achieve deep sedation or can lead to or easily induce a state of general anesthesia | - | - |
| *Propofol* | | | | | |
|  | Endoscopists and nurses participating in NAAP should undergo a training course | ESGE^17^ | GI endoscopists and registered nurses are adequate candidates for NAAP training courses. Previous experience in intensive care medicine or in anesthesia is desirable for the physician who is responsible for NAAP | Strong | Low |
|  |  | DSRPGSA^8^ | The doctor responsible for the examination has the overall responsibility for sedation and must have completed a theoretical and practical training course in propofol sedation  Nurses who help with, or perform sedation with propofol, must also have received structured, relevant training ie theoretical and practical training in propofol sedation and airway management | - | - |
|  |  | AGA^4^ | Specialized training is required for the physician and nursing staff before instituting a propofol sedation program | - | - |
|  |  | CAG^21^ | Endoscopists are required to obtain training in the safe administration of propofol before using it in clinical practice. We strongly recommend that endoscopists seeking to use propofol in their practice should undergo:   - Certification in advanced cardiac life support, and - A preceptorship or formal course of instruction with an individual (such as an anesthesiologist) who is familiar with propofol use | - | - |
|  |  | ASGE^19^ | NAPS should be administered by personnel specifically trained in its administration who should have expertise in airway management and must be present continuously during its use | - | - |
|  |  | ASGE^23^ | NAAP requires the acquisition of skills and abilities that are distinct and apart from those necessary for standard sedation. The training program should provide both didactic and practical, hands-on learning experiences | Strong | 1A |
|  |  | ISDE^27^ | Structured educational courses for NAAP are mandatory. Periodical retraining programs (at 2-3 year intervals) are strongly recommended | - | - |
|  | Physicians responsible for NAAP should have intensive care or anesthesia experience | ESGE^17^ | GI endoscopists and registered nurses are adequate candidates for NAAP training courses. Previous experience in intensive care medicine or in anesthesia is desirable for the physician who is responsible for NAAP | Strong | Low |
|  | Basic resuscitation skills | DSRPGSA^8^ | Staff managing or assisting in propofol sedation must be involved in this form of sedation on a regular basis and all must be trained in basic resuscitation | - | - |
|  |  | ASGE^23^ | Basic life support or advanced cardiac life support certification is required | Intermediate | 2A |
|  | Ability to manage complications | DSRPGSA^8^ | The doctor in question must also be trained in airway management and be able to handle any complications in cooperation with the NAPS nurse | - | - |
|  |  | CAG^21^ | Any ‘physician-nurse’ team involved in propofol use should have the requisite training to deal with significant respiratory depression | - | - |
|  |  | ASGE^19^ | Personnel should have the ability to rescue a patient who becomes unresponsive or unable to protect his or her airway or who loses spontaneous respiratory or cardiovascular function | - | - |
|  | ACLS training | CAG^21^ | Endoscopists are required to obtain training in the safe administration of propofol before using it in clinical practice. We strongly recommend that endoscopists seeking to use propofol in their practice should undergo:   - Certification in advanced cardiac life support, and - A preceptorship or formal course of instruction with an individual (such as an anesthesiologist) who is familiar with propofol use | - | - |
|  |  | ASGE^19^ | For propofol use during endoscopy:   - …at least 1 person who is qualified in advanced life support skills (ie airway management, defibrillation, and the use of resuscitative medications) | - | - |
|  |  | ASGE^23^ | Basic life support or advanced cardiac life support certification is required | Intermediate | 2A |
|  |  | ISDE^27^ | Personnel trained in ACLS should always be present in the endoscopic area | - | - |
|  | A life support team does not need to be immediately available if the endoscopy team has ACLS training | ESGE^17^ | There is no evidence that quick availability of a life support team is required for propofol administration. We do not recommend compulsory availability of a life support team if propofol is administered in the presence of a person trained in ACLS | C | 2+ |
|  | Skills in airway management | DSRPGSA^8^ | The doctor in question must also be trained in airway management and be able to handle any complications in cooperation with the NAPS nurse   - Before assuming unaided responsibility for NAPS sedation, the doctor must participate in a “NAPS course for doctors,” document practical skills in airway management supervised by anaesthetics staff, observed NAPS sedation and be thoroughly familiar with contraindications and the selection of patients for NAPS as well as current guidelines and instructions   Nurses who help with, or perform sedation with propofol, must also have received structured, relevant training ie theoretical and practical training in propofol sedation and airway management   - Endsocopy nurses who are required to perform propofol sedation must undergo 6 weeks of supervised theoretical and practical training | - | - |
|  |  | CAG^21^ | The endoscopist and nursing staff should be competent in airway management, as per the advanced cardiac life support guidelines | - | - |
|  |  | ASGE^19^ | NAPS should be administered by personnel specifically trained in its administration who should have expertise in airway management and must be present continuously during its use | - | - |
|  |  | ASGE^23^ | Individuals administering propofol should be proficient in the management of upper and lower airway complications, including manual techniques for re-establishing airway patency, use of oral and nasal airway devices, and proper bag-mask ventilation | Intermediate | 2A |
|  |  | ASGE^1^ | For deep sedation (including all uses of propofol), the endoscopist must have the ability to rescue the patient from general anesthesia, including managing a compromised airway | - | - |
|  |  | ISDE^27^ | Training in endotracheal intubation is not required for NAAP. Personnel providing NAAP must be trained to deal with significant respiratory depression in order to maintain airways patency; anesthesiologist support must be promptly available | - | - |
| *Sedation practice in general* | | | | | |
|  | Formal training in sedation | GSGMD^18^ | As part of quality assurance, we recommend that physicians and non-physician ancillary personnel should participate in specifically designed training for sedation | A | 5 |
|  |  | GESA^12^ | It is recommended that non-anesthetist medical or dental practitioners wishing to provide procedural sedation and/or analgesia have received a minimum of 3 months full time equivalent supervised training in procedural sedation and/or analgesia and anesthesia or similar approved course. Longstanding clinical experience may be deemed equivalent to a formal period of training | - | - |
|  |  | ASGE^19^ | We recommend that providers undergo specific training in the administration of endoscopic sedation and possess the skills necessary for the diagnosis and management of sedation-related adverse events, including rescue from a level of sedation deeper than that intended |  | High |
|  |  | SSGE^16^ | All endoscopy team members involved in sedation must be certified in both theoretical and practical sedation techniques | D | 4 |
|  |  | SAGES^6^ | An RN must have documented competence to administer conscious sedation and to assist in any support or resuscitation measures as required | - | - |
|  | Knowledge of sedative agents | GSGMD^18^ | We recommend that the physician who performs and is responsible for sedation should be experienced in intensive care medicine. He or she should be trained and proficient in the use of sedatives and analgesics. This includes knowledge, recognition, and treatment of expected side effects, including cardiopulmonary resuscitation, maintaining upper airway patency, intubation, and assisted ventilation | A | 5 |
|  |  | AGA^4^ | The endoscopist should be familiar with the pharmacokinetic and pharmacodynamics properties as well as potential drug-drug interactions of all agents used for sedation and reversal. An understanding of the time to peak effect is especially important to avoid oversedation during the induction phase of sedation | - | - |
|  |  | ASGH^5^ | The specially trained nurses must be familiar with the agent administered, be able to maintain respiration when complications occur or during the transition from deep sedation to general anesthesia, and be able to handle cardiovascular side effects or complications caused by the agent administered | - | - |
|  |  | ASGE^13^ | Individuals directing sedation should be specifically trained in endoscopic sedation, including the modes of action and adverse events of the sedative agents being used. | - | - |
|  |  | ASGE^1^ | Competence in sedation includes the ability to recognize the various levels of sedation from anxiolysis to general anesthesia. The endoscopist must understand the pharmacology of each sedative they intend to use, as well as the appropriate reversal agents | - | - |
|  |  | SSGE^16^ | Theoretical includes:…knowledge of drugs used for sedation, pharmacological and pharmacodynamics characteristics, administration regimens, dosages, synergies, interactions, and side effects; drug preparation and administration mode | - | - |
|  | Experience in intensive care medicine | GSGMD^18^ | We recommend that the physician who performs and is responsible for sedation should be experienced in intensive care medicine. He or she should be trained and proficient in the use of sedatives and analgesics. This includes knowledge, recognition, and treatment of expected side effects, including cardiopulmonary resuscitation, maintaining upper airway patency, intubation, and assisted ventilation | A | 5 |
|  | Ability to recognize and manage complications, including the ability to rescue from a deeper than intended level of sedation | AGA^4^ | Personnel who administer sedation agents should possess the ability to recognize and rescue patients whose level of sedation becomes deeper than originally intended  A nurse or assistant with appropriate training in endoscopic sedation should be present throughout the endoscopic procedure. This individual should have an understanding of the stages of sedation, an ability to monitor and interpret the patient’s physiologic parameters, skills to initiate appropriate intervention in the event of a complication, and current certification in basic or advanced cardiac life support  Practitioners intending to produce a given level of sedation should be able to rescue patients whose level of sedation becomes deeper than initially intended. Thus, individuals who administer moderate or deep sedation must be competent to administer reversal agents, manage an airway, and provide advanced cardiac life support (ACLS) care | - | - |
|  |  | ASGH^5^ | The specially trained nurses must be familiar with the agent administered, be able to maintain respiration when complications occur or during the transition from deep sedation to general anesthesia, and be able to handle cardiovascular side effects or complications caused by the agent administered | - | - |
|  |  | ASGE^13^ | The staff administering sedation must have the knowledge and skills to recognize when the sedation level becomes deeper than planned and to manage and support patients’ cardiopulmonary response to sedation accordingly | - | - |
|  |  | ASGE^19^ | We recommend that providers undergo specific training in the administration of endoscopic sedation and possess the skills necessary for the diagnosis and management of sedation-related adverse events, including rescue from a level of sedation deeper than that intended | - | High |
|  |  | ASGE^1^ | Of paramount importance is the ability to recognize complications of sedation (chiefly cardiorespiratory depression) and be able to rescue the patient | - | - |
|  |  | SSGE^16^ | Recognize the various sedation levels and possess skills to rescue patients anytime from a deeper-than-intended level | - | - |
|  |  | GESA^12^ | Practitioners who administer sedative or analgesic drugs that alter the conscious state of a patient must be prepared to manage the following potential risks:   - Depression of protective airway reflexes and loss of airway patency - Depression of respiration - Depression of the cardiovascular system - Drug interactions or adverse reactions, including anaphylaxis - Individual variations in response to the drugs used, particularly in children, the elderly, and those with pre-existing medical disease - The possibility of deeper sedation or anesthesia being used to compensate for inadequate analgesia or local anesthesia - Risks inherent in the wide variety of procedures performed under procedural sedation and/or analgesia - Unexpected extreme sensitivity to the drugs used for procedural sedation and/or analgesia which may result in unintended loss of consciousness, and respiratory or cardiovascular depression   The practitioner administering procedural sedation and/or analgesia requires sufficient training to be able to:   - Understand the actions of the drugs being administered, and be able to modify the technique appropriately in patients of different ages, or in the case of concurrent drug therapy or disease processes - Monitor the patient’s level of consciousness and cardiorespiratory status - Detect and manage appropriately any complications arising from sedation | - | - |
|  | Basic resuscitation skills | GSGMD^18^ | We recommend that the physician who performs and is responsible for sedation should be experienced in intensive care medicine. He or she should be trained and proficient in the use of sedatives and analgesics. This includes knowledge, recognition, and treatment of expected side effects, including cardiopulmonary resuscitation, maintaining upper airway patency, intubation, and assisted ventilation | A | 5 |
|  |  | AGA^4^ | A nurse or assistant with appropriate training in endoscopic sedation should be present throughout the endoscopic procedure. This individual should have an understanding of the stages of sedation, an ability to monitor and interpret the patient’s physiologic parameters, skills to initiate appropriate intervention in the event of a complication, and current certification in basic or advanced cardiac life support | - | - |
|  |  | ASGE^13^ | All staff with clinical responsibilities must have Basic Life Support (BLS) certification | - | - |
|  |  | SSGE^16^ | Have the necessary skills for airway management and certification of basic life support | - | - |
|  |  | SAGES^6^ | Staff members should be appropriately trained in resuscitative efforts. They should provide documentation of certification in courses such as basic cardiac life support and advanced cardiac life support (ACLS)…an assistant trained at least in basic cardiac life support should be present during all procedures to monitor the patient | - | - |
|  | Skills in airway management | GSGMD^18^ | We recommend that the physician who performs and is responsible for sedation should be experienced in intensive care medicine. He or she should be trained and proficient in the use of sedatives and analgesics. This includes knowledge, recognition, and treatment of expected side effects, including cardiopulmonary resuscitation, maintaining upper airway patency, intubation, and assisted ventilation | A | 5 |
|  |  | AGA^4^ | At least one member of a sedation team should be certified in advanced cardiac life support and capable of establishing an airway and providing positive-pressure ventilation  All endoscopists should possess current certification in advanced cardiac life support (or its equivalent) and should be capable of providing respiratory support for patients with apnea and upper airway obstruction. This includes use of jaw thrust and chin-lift maneuvers, oral or nasal airway, and bag-mask ventilation | - | - |
|  |  | GESA^12^ | A medical or dental practitioner who is skilled in airway management and cardiopulmonary resuscitation must be present whenever procedural sedation and/or analgesia are administered. | - | - |
|  |  | SSGE^16^ | Have the necessary skills for airway management and certification of basic life support | - | - |
|  | ACLS training | AGA^4^ | A nurse or assistant with appropriate training in endoscopic sedation should be present throughout the endoscopic procedure. This individual should have an understanding of the stages of sedation, an ability to monitor and interpret the patient’s physiologic parameters, skills to initiate appropriate intervention in the event of a complication, and current certification in basic or advanced cardiac life support  At least one member of a sedation team should be certified in advanced cardiac life support and capable of establishing an airway and providing positive-pressure ventilation  All endoscopists should possess current certification in advanced cardiac life support (or its equivalent) and should be capable of providing respiratory support for patients with apnea and upper airway obstruction. | - | - |
|  |  | SSGE^9^ | Personnel in charge of monitoring the vital signs and sedation of the patient must be trained in advanced cardiopulmonary resuscitation | D | 5 |
|  |  | ASGE^13^ | At least one individual with Advanced Cardiac Life Support (ACLS) certification must be present in the unit when patients are present | - | - |
|  |  | GESA^12^ | The proceduralist must have advanced life support skills and training | - | - |
|  |  | SAGES^6^ | Staff members should be appropriately trained in resuscitative efforts. They should provide documentation of certification in courses such as basic cardiac life support and advanced cardiac life support (ACLS). An ACLS-certified provider must accompany all sedated patients throughout their stay. | - | - |

ACG = American College of Gastroenterology; AGA = American Gastroenterological Association; ASGE = American Society for Gastrointestinal Endoscopy; ASGH = Austrian Society of Gastroenterology and Hepatology; BSG = British Society of Gastroenterology; CAG = Canadian Association of Gastroenterology; CCO = Cancer Care Ontario; CSGNA = Canadian Society of Gastroenterology Nurses and Associations; DSRPGSA = Danish Secretariat for Reference Programmes for Gastroenterology, Surgery and Anaesthetics; EC = European Commission; ESGE = European Society of Gastrointestinal Endoscopy; ESGENA = European Society of Gastrointestinal Endoscopy Nurses and Associates; FSDE = French Society of Digestive Endoscopy; GESA = Gastroenterological Society of Australia; GSGDMD = German Society for Gastroenterology, Digestive and Metabolic Diseases; ISDE = Italian Society of Digestive Endoscopy; JAG = Joint Advisory Group; SAGES = Society of American Gastrointestinal and Endoscopic Surgeons; SGNA = Society of Gastroenterology Nurses and Associates; SSGE = Spanish Society of Gastrointestinal Endoscopy

**Appendix 1.** Sources searched and search strategy

| **PubMed Search Strategy** | |
| --- | --- |
| (colonoscopy [mh] OR colonoscop*[tw] OR gastroscopy[mh] OR gastroscop*[tw] OR endoscopy[mh] or endoscop*) AND (Practice Guideline[ptyp] AND ("2015/10/31"[PDAT] : "2017/12/31"[PDAT]) AND English[lang]) | |
| **Guideline databases and websites** | **Search terms** |
| **Guideline databases and developers:**   - Standards and Guidelines Evidence Directory of Cancer Guidelines - Agency for Healthcare Research and Quality National Guideline Clearing House - National Institute for Health and Care Excellence - International Guideline Library - Canadian Medical Association InfoBase   **Websites:**   - Canadian Association of Gastroenterology - Canadian Anesthesiologists’ Society - Canadian Society of Gastroenterology Nurses and Associates - Ontario Association of Gastroenterology - American Gastroenterological Association - American Society for Gastrointestinal Endoscopy - Society of American Gastrointestinal and Endoscopic Surgeons - American College of Gastroenterology - Society of Gastroenterology Nurses and Associates - American Society of Anesthesiologists - Gastroenterological Society of Australia - British Society of Gastroenterology - Joint Advisory Group on GI Endoscopy (UK) - Spanish Society of Gastrointestinal Endoscopy - Endoscopy Section of the Netherlands Society of Gastroenterology - European Society of Gastrointestinal Endoscopy - European Society of Anesthesiology - World Gastroenterology Organization - Canadian provincial & territorial ministries of health & cancer agencies | 1. Colonoscopy  2. Gastroscopy  3. Endoscopy  4. Sedation |

**Appendix 2.** Articles excluded through full-text screening

***Reason for exclusion: Patient population out of scope (n=3)***

1. Canadian Agency for Drugs and Technologies in Health. Anaesthetic agents in pregnant women undergoing non-obstetric surgical or endoscopic procedures: a review of the safety and guidelines. Ottawa (ON): Canadian Agency for Drugs and Technologies in Health; 2015 [cited Feb 2017]. Available from: <https://www.cadth.ca/sites/default/files/pdf/htis/june-2015/RC0664%20Anaesthesia%20Safety%20in%20Pregnancy%20Final.pdf>.
2. Qureshi WA, Rajan E, Adler DG, Davila RE, Hirota WK, Jacobson BC, et al. ASGE Guideline: Guidelines for endoscopy in pregnant and lactating women. Gastrointest Endosc. 2005;61(3):357-62.
3. Gilinsky NH, Muthunayagam N. Gastrointestinal endoscopy in pregnant and lactating women: emerging standard of care to guide decision-making. Obstet Gynecol Surv. 2006;61(12):791-9

***Reason for exclusion: Not related to routine gastrointestinal endoscopy (n=14)***

1. Burlingame B DB, Link T, Ogg MJ, Spruce L, Spry C, Van Wicklin SA, Wood A. Guideline for care of the patient receiving moderate sedation/analgesia. guidelines for perioperative practice. 2017;1.
2. Dobson G, Chong M, Chow L, Flexman A, Kurrek M, Laflamme C, et al. Guidelines to the practice of anesthesia - revised edition 2017. Can J Anaesth. 2017;64(1):65-91. Available from: http://www.cas.ca/English/Page/Files/97_Guidelines-2017.pdf
3. Albeniz, E.,Pellise, M.,Gimeno Garcia, A. Z., et al. Clinical guidelines for endoscopic mucosal resection of non-pedunculated colorectal lesions. Gastroenterol Hepatol. 2018. 41:175-190
4. Inoue, H.,Shiwaku, H.,Iwakiri, K., et al. Clinical practice guidelines for peroral endoscopic myotomy. Dig Endosc. 2018. 30:563-579
5. Mayeaux, E. J., Jr.,Novetsky, A. P.,Chelmow, et al. ASCCP Colposcopy Standards: Colposcopy Quality Improvement Recommendations for the United States. J Low Genit Tract Dis. 2017. 21:242-248
6. Polkowski, M.,Jenssen, C.,Kaye, P., et al. Technical aspects of endoscopic ultrasound (EUS)-guided sampling in gastroenterology: European Society of Gastrointestinal Endoscopy (ESGE) Technical Guideline - March 2017. Endoscopy. 2017. 49:989-1006
7. Rondonotti, E.,Spada, C.,Adler, S., et al. Small-bowel capsule endoscopy and device-assisted enteroscopy for diagnosis and treatment of small-bowel disorders: European Society of Gastrointestinal Endoscopy (ESGE) Technical Review. Endoscopy. 2018. 50:423-446
8. Teoh, A. Y. B.,Dhir, V.,Kida, M., et al. Consensus guidelines on the optimal management in interventional EUS procedures: results from the Asian EUS group RAND/UCLA expert panel. Gut. 2018. 67:1209-1228
9. Wani, S.,Keswani, R. N.,Petersen, B., et al. Training in EUS and ERCP: standardizing methods to assess competence. Gastrointest Endosc. 2018. 87:1371-1382
10. Waxman, A. G.,Conageski, C.,Silver, M. I., et al. ASCCP Colposcopy Standards: How Do We Perform Colposcopy? Implications for Establishing Standards. J Low Genit Tract Dis. 2017. 21:235-241
11. Wentzensen, N.,Massad, L. S.,Mayeaux, E. J., et al. Evidence-Based Consensus Recommendations for Colposcopy Practice for Cervical Cancer Prevention in the United States. J Low Genit Tract Dis. 2017. 21:216-222
12. Yamamoto, H.,Ogata, H.,Matsumoto, T., et al. Clinical Practice Guideline for Enteroscopy. Dig Endosc. 2017. 29:519-546
13. Espinet Coll, E.,Lopez-Nava Breviere, G., et al. Spanish Consensus Document on Bariatric Endoscopy. Part 1. General considerations. Rev Esp Enferm Dig. 2018. 110:386-399
14. Academy of Medical Royal Colleges. Safe Sedation Practice for Healthcare Procedures Standards and Guidance 2013 [Available from: https://www.rcoa.ac.uk/system/files/PUB-SafeSedPrac2013.pdf.

***Reason for exclusion: Did not address study aims (n=27)***

1. Bretthauer M, Aabakken L, Dekker E, Kaminski MF, Rosch T, Hultcrantz R, et al. Requirements and standards facilitating quality improvement for reporting systems in gastrointestinal endoscopy: European Society of Gastrointestinal Endoscopy (ESGE) Position Statement. Endoscopy. 2016;48(3):291-4.
2. Rutter MD, Senore C, Bisschops R, Domagk D, Valori R, Kaminski MF, et al. The European Society of Gastrointestinal Endoscopy Quality Improvement Initiative: developing performance measures. United European Gastroenterol J. 2016;4(1):30-41.
3. Ben-Menachem T, Decker GA, Early DS, Evans J, Fanelli RD, Fisher DA, et al. Adverse events of upper GI endoscopy. Gastrointest Endosc. 2012;76(4):707-18.
4. Armstrong D, Barkun A, Bridges R, Carter R, de Gara C, Dube C, et al. Canadian Association of Gastroenterology consensus guidelines on safety and quality indicators in endoscopy. Can J Gastroenterol. 2012;26(1):17-31.
5. Romagnuolo J, Enns R, Ponich T, Springer J, Armstrong D, Barkun AN. Canadian credentialing guidelines for colonoscopy. Can J Gastroenterol. 2008;22(1):17-22.
6. Ponich T, Enns R, Romagnuolo J, Springer J, Armstrong D, Barkun AN. Canadian credentialing guidelines for esophagogastroduodenoscopy. Can J Gastroenterol. 2008;22(4):349-54.
7. Fisher DA, Maple JT, Ben-Menachem T, Cash BD, Decker GA, Early DS, et al. Complications of colonoscopy. Gastrointest Endosc. 2011;74(4):745-52.
8. Ikenberry SO, Anderson MA, Banerjee S, Baron TH, Dominitz JA, Gan SI, et al. Endoscopy by nonphysicians. Gastrointest Endosc. 2009;69(4):767-70.
9. Gastroenterological Society of Australia. Standards for endoscopic facilities and services. Victoria, Australia: Gastroenterological Society of Australia; 2006 [cited Feb 2017]. Available from: <http://membes.gesa.org.au/membes/files/Clinical%20Guidelines%20and%20Updates/Endoscopy_Standards.pdf>.
10. Dumonceau JM, Riphaus A, Beilenhoff U, Vilmann P, Hornslet P, Aparicio JR, et al. European curriculum for sedation training in gastrointestinal endoscopy: position statement of the European Society of Gastrointestinal Endoscopy (ESGE) and European Society of Gastroenterology and Endoscopy Nurses and Associates (ESGENA). Endoscopy. 2013;45(6):496-504.
11. Hori Y. Granting of privilege for gastrointestinal endoscopy. Surg Endosc. 2008;22(5):1349-52.
12. Borgaonkar MR, Hookey L, Hollingworth R, Kuipers EJ, Forster A, Armstrong D, et al. Indicators of safety compromise in gastrointestinal endoscopy. Can J Gastroenterol. 2012;26(2):71-8.
13. Vargo JJ, DeLegge MH, Feld AD, Gerstenberger PD, Kwo PY, Lightdale JR, et al. Multisociety sedation curriculum for gastrointestinal endoscopy. Gastrointest Endosc. 2012;76(1):e1-25.
14. Adler DG, Bakis G, Coyle WJ, DeGregorio B, Dua KS, Lee LS, et al. Principles of training in GI endoscopy. Gastrointest Endosc. 2012;75(2):231-5.
15. Rembacken B, Hassan C, Riemann JF, Chilton A, Rutter M, Dumonceau JM, et al. Quality in screening colonoscopy: position statement of the European Society of Gastrointestinal Endoscopy (ESGE). Endoscopy. 2012;44(10):957-68.
16. Rizk MK, Sawhney MS, Cohen J, Pike IM, Adler DG, Dominitz JA, et al. Quality indicators common to all GI endoscopic procedures. Gastrointest Endosc. 2015;81(1):3-16.
17. Rex DK, Schoenfeld PS, Cohen J, Pike IM, Adler DG, Fennerty MB, et al. Quality indicators for colonoscopy. Gastrointest Endosc. 2015;81(1):31-53.
18. Park WG, Shaheen NJ, Cohen J, Pike IM, Adler DG, Inadomi JM, et al. Quality indicators for EGD. Gastrointest Endosc. 2015;81(1):17-30.
19. Bohlander S, Grand A, Kelsey L, Miller LD, Herron-Rice L, Taylor C, et al. Standards of professional performance and standards of practice for the gastroenterology and/or endoscopy setting. Gastroenterol Nurs. 2005;28(5):422-7.
20. Faigel DO, Cotton PB. The London OMED position statement for credentialing and quality assurance in digestive endoscopy. Endoscopy. 2009;41(12):1069-74.
21. Vargo JJ, Ahmad AS, Aslanian HR, Buscaglia JM, Das AM, Desilets DJ, et al. Training in patient monitoring and sedation and analgesia. Gastrointest Endosc. 2007;66(1):7-10.
22. Faulx AL, Lightdale JR, Acosta RD, Agrawal D, Bruining DH, Chandrasekhara V, et al. Guidelines for privileging, credentialing, and proctoring to perform GI endoscopy. Gastrointest Endosc. 2017;85(2):273-81.
23. BC Cancer Agency. Colon Screening Program Colonoscopy Standards. Vancouver (BC): BC Cancer Agency; 2016 [cited Feb 2017]. Available from: <http://www.bccancer.bc.ca/screening/Documents/COLON_GuidelinesManual-StandardsColonoscopy.pdf>.
24. Quality indicators in colonoscopy. The colonoscopy procedure. Rev Esp Enferm Dig. 2018. 110:316-326
25. Beg, S.,Ragunath, K.,Wyman, A., et al. Quality standards in upper gastrointestinal endoscopy: a position statement of the British Society of Gastroenterology (BSG) and Association of Upper Gastrointestinal Surgeons of Great Britain and Ireland (AUGIS). Gut. 2017. 66:1886-1899
26. Chan, F. K. L.,Goh, K. L.,Reddy, et al. Management of patients on antithrombotic agents undergoing emergency and elective endoscopy: joint Asian Pacific Association of Gastroenterology (APAGE) and Asian Pacific Society for Digestive Endoscopy (APSDE) practice guidelines. Gut. 2018. 67:405-417
27. Kaminski, M. F.,Thomas-Gibson, S.,Bugajski, M., et al. Performance measures for lower gastrointestinal endoscopy: a European Society of Gastrointestinal Endoscopy (ESGE) Quality Improvement Initiative. Endoscopy. 2017. 49:378-397

***Reason for exclusion: Document type out of scope (n=5)***

1. Alberta Health Services. Procedural sedation. Alberta, Canada: Alberta Health Services; 2016 [cited Feb 2017]. Available from: <https://extranet.ahsnet.ca/teams/policydocuments/1/clp-procedural-sedation-policy.pdf>.
2. Canadian Agency for Drugs and Technologies in Health. Sedation and anesthesia options for diagnostic procedures: a review of clinical effectiveness and guidelines. Ottawa (ON): Canadian Agency for Drugs and Technologies in Health; 2015 [cited Feb 2017]. Available from: <https://www.cadth.ca/sites/default/files/pdf/htis/may-2015/RC0654%20MRI%20Sedation%20Final.pdf>.
3. Moulton K, Cunningham J. Respiratory and cardiac monitoring for patients receiving propofol for procedural sedation: guidelines. Ottawa (ON): Canadian Agency for Drugs and Technologies in Health; 2010 [cited Feb 2017]. Available from: <https://www.cadth.ca/sites/default/files/pdf/K0136_Respiratory_and_Cardiac_Monitoring_for_Propofol_final.pdf>.
4. Kamel C, Moulton K, Cunningham J. Short-acting agents and dissociative agents for conscious sedation during endoscopy procedures: systematic review of clinical and cost-effectiveness and guidelines. Ottawa (ON): Canadian Agency for Drugs and Technologies in Health; 2010 [cited Feb 2017]. Available from: <http://www.cadth.ca/index.php/en/hta/reports-publications/search?&type=34>.
5. Cohen J, Pike IM. Defining and measuring quality in endoscopy. Am J Gastroenterol. 2015;110(1):46-7.

***Reason for exclusion: Could not locate full-text document (n=1)***

1. American Society of Anesthesiologists. Statement on anesthesia care for endoscopic procedures [Internet]. American Society of Anesthesiologists; 2014 [cited 2017 Feb 27]. Available from: http://www.asahq.org/~/media/Sites/ASAHQ/Files/Public/Resources/standards- guidelines/statement-on-anesthesia-care-for-endoscopic-procedures.pdf

**References**

1. Ensuring competence in endoscopy. American Society for Gastrointestinal Endoscopy, 2006. at <http://s3.gi.org/physicians/EnsuringCompetence.pdf>.)

2. British Society of Gastroenterology. Complications of gastrointestinal endoscopy. British Society of Gastroenterology 2006. at <http://www.bsg.org.uk/pdf_word_docs/complications.pdf>.)

3. Napolean B, Ponchon T, Lefebvre RR, et al. French Society of Digestive Endoscopy (SFED) Guidelines on performing a colonoscopy. Endoscopy 2006;38:1152-5.

4. Cohen LB, Delegge MK, Aisenberg J, et al. AGA Institute Review of Endoscopic Sedation. Gastroenterology 2007;133:675-701.

5. Schreiber F. The Working Group of Endoscopy, Austrian Society of Gastroenterology and Hepatology (ÖGGH). Austrian Society of Gastroenterologya and Hepatology (ÖGGH)— Guidelines on sedation and monitoring during gastrointestinal endoscopy. Endoscopy 2007;39:259-62.

6. Heneghan S, Myers J, Fanelli R, W R. Society of American Gastrointestinal Endoscopic Surgeons (SAGES) guidelines for office endoscopic services. Surg Endosc 2009;23:1125-9.

7. Jain R, Ikenberry SO, Anderson MA, et al. Minimum staffing requirements for the performance of GI endoscopy. Gastrointestinal endoscopy 2010;72:469-70.

8. Danish Secretariat for Reference Programmes for Gastroenterology, Surgery and Anaesthetics. Propofol sedation for gastroenterological, endoscopic procedures performed by non-anesthetically trained personnel - and associated training. Danish Secretariat for Reference Programmes for Gastroenterology, Surgery and Anaesthetics, 2011. at <http://www.esgena.org/assets/downloads/pdfs/guidelines/2011_danish_propofol_guideline.pdf>.)

9. Jover R, Herraiz M, Alarcón O, et al. Clinical practice guidelines: quality of colonoscopy in colorectal cancer screening. Endoscopy 2012;44:444-51.

10. Valori R, Rey J, Atkin W, et al. European guidelines for quality assurance in colorectal cancer screening and diagnosis. –Quality assurance in endoscopy in colorectal cancer screening and diagnosis. Endoscopy 2012;44:SE88-SE105.

11. Early DS, Acosta RD, Chandrasekhara V, et al. Modifications in endoscopic practice for the elderly. Gastrointestinal endoscopy 2013;78:1-7.

12. Australian and New Zealand College of Anaesthetists (ANZCA). Guidelines on Sedation and/or Analgesia for Diagnostic and Interventional Medical, Dental or Surgical Procedures. 2014. at <https://www.anzca.edu.au/documents/ps09-2014-guidelines-on-sedation-and-or-analgesia>.)

13. Calderwood AH, Chapman FJ, Cohen J, et al. Guidelines for safety in the gastrointestinal endoscopy unit. Gastrointestinal endoscopy 2014;79:363-72.

14. Guideline for colonoscopy quality assurance in Ontario Cancer Care Ontario, 2013. at <https://www.cancercare.on.ca/common/pages/UserFile.aspx?fileId=33457>.)

15. Tinmouth J, Kennedy EB, Baron D, et al. Colonoscopy quality assurance in Ontario: systematic review and clinical practice guideline. Canadian Journal of Gastroenterology and Hepatology 2014;28:251-74.

16. Igea F, Casellas JA, González-Huix F, et al. Sedation for gastrointestinal endoscopy. Clinical practice guidelines of the Sociedad Española de Endoscopia Digestiva. Rev Esp Enferm Dig 2014;106:195-211.

17. Dumonceau J-M, Riphaus A, Schreiber F, et al. Non-anesthesiologist administration of propofol for gastrointestinal endoscopy: European Society of Gastrointestinal Endoscopy, European Society of Gastroenterology and Endoscopy Nurses and Associates Guideline–Updated June 2015. Endoscopy 2015;47:1175-89.

18. Riphaus A, Wehrmann T, Hausmann J, et al. Update S3-guideline:“sedation for gastrointestinal endoscopy” 2014 (AWMF-register-no. 021/014). Zeitschrift für Gastroenterologie 2016;54:58-95.

19. Early DS, Lightdale JR, Vargo JJ, et al. Guidelines for sedation and anesthesia in GI endoscopy. Gastrointestinal endoscopy 2018;87:327-37.

20. Dunkley I, Griffiths H, Follows R, et al. UK consensus on non-medical staffing required to deliver safe, quality-assured care for adult patients undergoing gastrointestinal endoscopy. British Medical Journal Publishing Group; 2019.

21. Byrne MF, Chiba N, Singh H, Sadowski DC, Gastroenterology CACotCAo. Propofol use for sedation during endoscopy in adults: a Canadian Association of Gastroenterology position statement. Canadian Journal of Gastroenterology and Hepatology 2008;22:457-9.

22. SGNA Practice Committee. Statement on the use of sedation and analgesia in the gastrointestinal endoscopy setting. Gastroenterology nursing: the official journal of the Society of Gastroenterology Nurses and Associates 2008;31:249.

23. Vargo JJ, Cohen LB, Rex DK, Kwo PY. Position statement: nonanesthesiologist administration of propofol for GI endoscopy. The American journal of gastroenterology 2009;104:2886.

24. American Society of Gastrointestinal Endoscopy, American College of Gastroenterology, American Gastroenterological Association. Universal adoption of capnography for moderate sedation in adults undergoing upper endoscopy and colonoscopy has not been show to improve patient safety for clinical outcomes and significantly increases costs for moderate sedation American Society of Gastrointestinal Endoscopy, 2012. at <https://www.asge.org/docs/default-source/education/practice_guidelines/doc-90dc9b63-593d-48a9-bec1-9f0ab3ce946a.pdf?sfvrsn=6>.)

25. Canadian Society of Gastroenterology Nurses and Associates. Procedural sedation: position statement for the role of the registered nurse. Canadian Society of Gastroenterology Nurses and Associates, 2015. at <https://colibri-production-app.s3.amazonaws.com/sites/555e068a83781212ec01929d/assets/55f6bec64a2c09a49800ead0/Procedural_Sedation_position_statement_and_role_of_the_RN_April_11_2015.pdf>.)

26. Society of Gastroenterology Nurses and Associates. Minimum registered nurse staffing for patient care in the gastroenterology setting. Society of Gastroenterology Nurses and Associates, 2016. at [https://www.sgna.org/Portals/0/Minimum RN Staffing_FINAL.pdf](https://www.sgna.org/Portals/0/Minimum%20RN%20Staffing_FINAL.pdf).)

27. Conigliaro R, Fanti L, Manno M, Brosolo P. Italian Society of Digestive Endoscopy (SIED) position paper on the non-anaesthesiologist administration of propofol for gastrointestinal endoscopy. Digestive and Liver Disease 2017;49:1185-90.
